# Phenolic-Enriched Pullulan Coatings: Molecular Interactions and Functional Properties for Active Food Packaging Applications

**DOI:** 10.1021/acsomega.6c00375

**Published:** 2026-03-02

**Authors:** Athira John, Klementina Pušnik Črešnar, David Hvalec, Maša Knez Marevci, Dimitrios N. Bikiaris, Lidija Fras Zemljič

**Affiliations:** † Faculty of Mechanical Engineering, 54765 University of Maribor, Smetanova Ulica 17, Maribor 2000, Slovenia; ‡ Faculty of Chemistry and Chemical Engineering, 200917 University of Maribor, Smetanova 17, Maribor 2000, Slovenia; § Faculty of Medicine, University of Maribor, Taborska Ulica 8, Maribor 2000, Slovenia; ∥ Laboratory of Polymer Chemistry and Technology, Department of Chemistry, 37782 Aristotle University of Thessaloniki, Thessaloniki, 541 24, Greece

## Abstract

Sustainable active coatings based on renewable polymers are increasingly sought for food-packaging applications; however, surface-applicable colloidal coating systems remain markedly underexplored compared to conventional bulk films. In practical applications, coatings are applied as liquid colloidal dispersions, which subsequently form solid films at the food–material interface, where their functionality is ultimately expressed. A predictive understanding of coating performance, therefore, critically depends on a comprehensive characterization of both the colloidal state and the resulting film, an aspect that is often underestimated in current formulation-driven approaches. In this study, we report pullulan-based colloidal coatings functionalized with polyphenol-rich yerba mate (YE) and chestnut wood (WE) extracts, obtained via green ultrasound-assisted aqueous extraction. Distinct from conventional cast-film-centric studies, this work adopts a structure–property-driven strategy, systematically linking the physicochemical and colloidal properties of the liquid formulations to the interfacial, structural, and functional properties of the formed films. Such an integrated approach enables informed optimization and rational manipulation of coating performance already at the formulation stage, rather than relying on empirical surface deposition alone. HPLC analysis of the extracts identified chlorogenic, caffeic, rutin, and ellagic acids as the dominant phenolics governing bioactivity. The incorporation of YE and WE into pullulan significantly enhanced colloidal stability (ζ ≈ −25 mV; PDI ≈ 0.16) in dispersion, while, upon film formation, it reduced the water contact angle (54.6° vs 65° for neat pullulan) and increased surface free energy by 26.3%, indicating improved interfacial performance. ATR-FTIR and XRD analyses confirmed noncovalent pullulan–polyphenol interactions while preserving the amorphous polymer structure. The resulting coatings exhibited effective UV shielding, strong antioxidant activity (near-complete radical scavenging within 60 min), and antibacterial efficacy against *Staphylococcus aureus* (12 ± 2.8 mm inhibition zone). Overall, this work demonstrates that a combined colloidal–film characterization framework is essential for the rational design of functional biopolymer coatings and highlights pullulan–polyphenol dispersions as promising, biodegradable, and multifunctional active layers for sustainable food-packaging applications.

## Graphical abstract

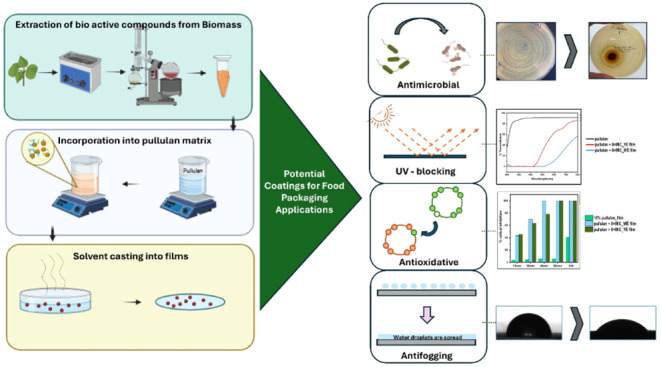

## 1 Introduction

In response to increasing consumer and regulatory demands for fresh, safe, and environmentally sustainable products, the role of food packaging has evolved beyond passive containment. Active packaging systems, which interact dynamically with food and its surrounding environment, have emerged as a key strategy to preserve quality, enhance safety, and extend shelf life.^1^ Unlike conventional packaging, active systems incorporate functional agents capable of scavenging oxygen, inhibiting microbial growth, or mitigating oxidative degradation. Recent advances in biopolymer-based and biodegradable materials have further accelerated the development of such technologies, offering sustainable alternatives to petrochemical-derived packaging.^2,3^

In parallel, functional coatings for food packaging have gained increasing attention as an attractive route toward lightweight, material-efficient, and surface-specific solutions. Such coatings must combine sustainability with the ability to form thin, uniform, and continuous films while maintaining desirable barrier and mechanical properties.^4^ Biopolymers including chitosan, alginate, proteins, and starch derivatives have therefore been widely explored due to their biodegradability, film-forming ability, and, in some cases, inherent antimicrobial or oxygen barrier properties.^5,6^ Among these materials, pullulan—a linear, water-soluble polysaccharide produced by *Aureobasidium pullulans* *—*stands out owing to its excellent film-forming capacity, outstanding oxygen barrier performance, edibility, and biodegradability. Structurally composed of maltotriose units linked by α-(1→6) bonds with internal α-(1→4) linkages, pullulan readily forms transparent and flexible coatings that effectively limit oxidative degradation in foods such as cheese, nuts, and baked goods^7–10^ (Figure 1).

**Figure 1:**
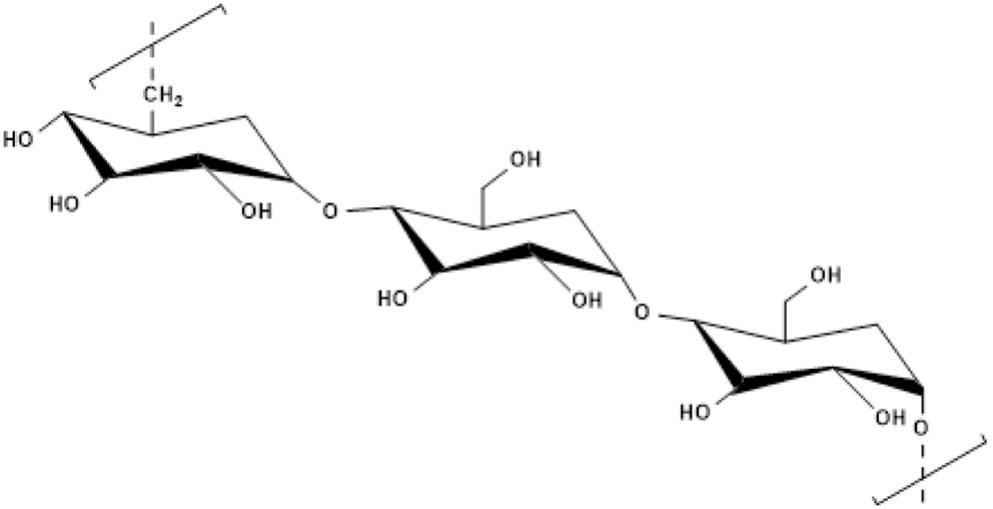
Structure of pullulan.

Despite these advantages, pullulan-based coatings remain largely passive and require functionalization to achieve active preservation. While previous studies have demonstrated their ability to reduce oxygen ingress and moisture loss, their antioxidant or antimicrobial performance is limited without the incorporation of bioactive agents.^11,12^ Consequently, recent research has focused on hybrid pullulan systems containing natural additives such as essential oils, polyphenolic extracts, and nanostructured fillers to simultaneously deliver barrier functionality and bioactivity.^8,13–15^ These composite systems are particularly attractive for coating technologies due to their compatibility with industrial deposition methods, including spraying, dipping, and roller coating.^16–19^

Among natural bioactive additives, yerba mate (*Ilex paraguariensis*) and chestnut wood (*Castanea* spp.) extracts have attracted considerable attention due to their high polyphenol content and pronounced antioxidant and antimicrobial activity. Yerba mate, rich in phenolic acids and flavonoids, has been shown to suppress microbial growth and oxidative spoilage in packaged foods.^20,21^ Its incorporation into starch-based films has yielded biodegradable materials with enhanced antioxidant, antibacterial, and even smart sensing properties.^22^ Similarly, chestnut wood extracts, abundant in tannins and flavonoids, are valued for their antimicrobial efficacy, biodegradability, and nontoxicity.^23^ When embedded in biopolymer matrices, these extracts improve mechanical strength, thermal stability, barrier properties, and antioxidant performance, as demonstrated for alginate- and chitosan-based films, as well as active packaging components such as antimicrobial sachets.^24–27^

While these studies clearly demonstrate the potential of yerba mate and chestnut wood extracts, they predominantly focus on bulk cast films, overlooking the fact that, in practical applications, coatings are applied as liquid formulations that subsequently form solid films at the food–material interface. As a result, the role of colloidal stability, rheology, interfacial properties, and formulation-level interactions remains insufficiently understood. This gap limits the predictive design and rational optimization of functional coatings for real-world applications.

In this work, we address this limitation by shifting from conventional bulk films to pullulan-based colloidal coating systems explicitly designed for surface applications. The novelty of this study lies in (i) the development of stable, spray- and dip-applicable pullulan–polyphenol colloidal formulations, and (ii) the use of green ultrasound-assisted aqueous extraction to preserve phenolic functionality while ensuring sustainability. Crucially, this work establishes systematic correlations between molecular interactions, colloidal stability, surface and interfacial properties, and the structure and performance of the resulting solid films. By integrating formulation science with coating-relevant characterization, we demonstrate how underutilized natural extracts can be translated into multifunctional, biodegradable coatings with antioxidant, antimicrobial, and UV-protective properties, advancing sustainable strategies for active food packaging.

## 2 Experimental Section

### 2.1 Materials

The pullulan was purchased from TCI Europe (Zwijndrecht, Belgium). The glycerol, acetic acid (≥99.8% (v/v)), potassium persulfate (K_2_S_2_O_8_, 99.99%), Folin–Ciocalten reagent, sodium­(V) carbonate (Na_2_CO_3_) and 2,2-diphenyl-1-picrylhydrazyl (DPPH) were obtained from Sigma-Aldrich (St. Louis, USA). The 2,2′-Azino-bis (3-ethylbenzothiazoline-6-sulfonic acid) (ABTS) was acquired from Thermo Scientific (Waltham, USA). The methanol (99.8%) was sourced from Pregl Chemicals (Mikropolo, Slovenia). The phosphate buffered saline (PBS, pH 7.2) was procured from Chemsolute Th. Geyer & Co KG (Renningen, Germany). The ultrapure water (resistivity 18.2 MΩ·cm at 25 °C, pH 6.9) was prepared using a Milli-Q purification system (Millipore Corporation, Bedford, USA). All the chemicals were used as received without further purification. The antimicrobial tests were performed externally at the National Laboratory of Health, Environment and Food (NLZOH), Maribor, Slovenia. As the testing was conducted externally, detailed information about the material procurement by NLZOH was not available

The analytical standards of the catechin, epicatechin, caffeic acid, quercetin, chlorogenic acid, p-coumaric acid, rutin trihydrate, gallic acid and ellagic acid were obtained from Sigma-Aldrich. To determine the phenolic compounds by HPLC, we used methanol (MeOH) with a purity ≥99.9% (Honeywell, Charlotte, NC, USA, LC-MS CHROMASOLV).

### 2.2 Methods

#### 2.2.1 Biomaterials̀ Extraction

In this study 20 g of chestnut wood (*Castanea sativa*) bark and yerba mate (*Ilex paraguariensis*) were processed to obtain the active components. The chestnut wood bark was delivered from Tanin Sevnica Kemična industrija d.d.o. and was already chopped into small pieces and dried, while the yerba mate was obtained from a local supermarket. Each material was crushed into a fine powder individually, using a mechanical grinder to ensure uniformity. For each material, the powdered sample was mixed with 150 mL of distilled water in a glass beaker to form a suspension.

Ultrasonic-assisted extraction (UAE) was employed to extract the target compounds from each material. The suspensions were subjected to ultrasonic treatment using an ultrasonic bath (Vevor ultrasonicator), operating at a frequency of 40 kHz and a power output of 200 W. The extraction process was carried out at room temperature for 2 h to ensure efficient extraction of the desired constituents. Following the extraction, each aqueous solution was filtered through a 0.45 μm membrane filter to remove any residual particulate matter. The filtrates were then concentrated using a rotary evaporator (Büchi Rotavapor R-300) under reduced pressure (100 bar), and a controlled temperature of 40 °C to prevent thermal degradation of the extracted compounds. The evaporation process continued until the solvent was removed completely, producing concentrated extracts for subsequent analysis.

#### 2.2.2 Preparation of Colloidal Dispersions

A 10% (w/v) macromolecular dispersion of pullulan (denoted as 10% Pullulan) was prepared by dissolving pullulan powder in Milli-Q water under continuous stirring for 24 h, at an ambient temperature to ensure complete dissolution and homogeneity. The resulting pullulan dispersion was stored at 5 °C until further use.

To prepare the macromolecular colloidal dispersions of the wood extracts (WE) and yerba mate extracts (YE), 3.75 and 7.50 g of the respective dry plant extracts were added to 100 mL of the previously prepared 10% pullulan solution. The pH of the pullulan solution was adjusted to 5.0 using dilute acetic acid prior to the addition of the extracts, to ensure the neutrality of the pullulan solution. The quantities of wood and yerba mate extracts were determined based eight times on the previously established minimal inhibitory concentration (MIC) for each extract. The prepared formulations of WE and YE are designated as Pullulan +8× MIC_WE and Pullulan +8× MIC_YE respectively.

The dry plant extracts solutions were prepared by dissolving 3.75 and 7.50 g of the wood and yerba mate extracts, respectively, in 100 mL Milli-Q water, and stirred for 24 h to ensure uniform dissolution. The solutions are annotated as 8× MIC_WE and 8× MIC_YE respectively. The final colloidal dispersions were allowed to equilibrate for 1 h at room temperature before further characterization or use in the subsequent experiments. All the procedures were conducted under aseptic conditions to prevent contamination.

#### 2.2.3 Preparation of Cast Films

The 10% Pullulan macromolecular solution and the Pullulan +8× MIC_WE and Pullulan +8× MIC_YE mixtures were then cast into freestanding films, to determine their potential as coatings. For this purpose, 25 mL of the above-prepared macromolecular colloidal dispersions, containing 5% glycerol (w/w, based on the total weight of the dispersion) as a plasticizer, were poured into 8 cm Petri dishes. The films were dried at room temperature for 48 h, to ensure complete solvent evaporation and uniform film formation. The dried films were peeled off carefully and stored in a desiccator at room temperature until further use.

All the sample notations used in this study are summarized in Table 1.

**Table 1:** Summary of Samples and Their Corresponding Notations Used in This Study^a^

Sample	Annotations
Pullulan powder	Pullulan
Wood extract powder	WE
Yerba mate extract powder	YE
10% Pullulan macromolecular solution	10% Pullulan
8 times MIC of WE dispersion	8× MIC_WE
8 times MIC of YE dispersion	8× MIC_YE
10% pullulan incorporated with 8 times MIC of WE	Pullulan +8× MIC_WE
10% pullulan incorporated with 8 times MIC of YE	Pullulan +8× MIC_YE
10% pullulan with 5% glycerol plasticizer solvent-casted film	10% Pullulan film
10% pullulan film incorporated with 8 times MIC of WE	Pullulan +8× MIC_WE film
10% pullulan film incorporated with 8 times MIC of YE	Pullulan +8× MIC_WE film

a MIC—minimal inhibitory concentration.

#### 2.2.4 Characterizations

##### 2.2.4.1 Pullulan and Bioextracts

*ATR-FTIR Spectroscopy* The attenuated total reflectance Fourier-transform infrared (ATR-FTIR) of the pullulan, YE and WE were recorded on a PerkinElmer Spectrum PerkinElmer Spectrum 3 spectrometer (PerkinElmer FTIR, Omega, Slovenia) with a PIKE GladiATR accessory (PIKE Technologies, Omega, Slovenia). All the spectra (32 scans at 4 cm^–1^ resolution, background, and the sample spectra, were obtained in the 400–4000 cm^–1^ wavenumber range) were recorded at room temperature. All the spectra presented here were baseline corrected and smoothed upon the measurement.

*Determination of the Total Phenolic Content (TPC) and Antioxidant Activities* The total phenolic content of the pullulan, wood extracts (WE) and yerba extracts (YE) was determined using the Folin-Ciocalteu assay. Twenty milligrams of extract was weighed into a 10 mL volumetric flask, and diluted to the mark with distilled water to prepare a 2 mg/mL solution. For the reaction, 0.5 mL of the extract solution was transferred into a test bottle, followed by the addition of 2.5 mL of Folin–Ciocalteu reagent (diluted 1:10 with water) and 2 mL of the Na_2_CO_3_ solution (75 g/L). The prepared samples were incubated in a water bath at 50 °C for 5 min, then cooled to room temperature. The absorbance was measured at 760 nm using a UV–vis spectrophotometer (Agilent Cary 60 instrument). A control sample was prepared by replacing the extract solution with distilled water and was used for spectrophotometer calibration. A gallic acid calibration curve (0.01–1 mg/mL) was employed to express the TPC of the examined samples. The findings were expressed as gallic acid equivalents (mg GAE/100 g dry weight).

*Antioxidant Activity* The antioxidant activity of the pullulan and extract samples was evaluated using two widely accepted radical scavenging assays: the 2,2′-azino-bis­(3-ethylbenzothiazoline-6-sulfonic acid) diammonium salt (ABTS^•^+^^) assay and the 2,2-diphenyl-1-picrylhydrazyl (DPPH^•^) assay. The absorbance measurements were performed using a probe-based UV–vis spectrophotometer (Cary 60, Agilent Technologies, USA), equipped with a fiber-optic immersion probe to facilitate direct, in-sample analysis.

*ABTS^•^+^^ Radical Scavenging Assay* The ABTS^•^+^^ radical cation was generated by reacting 7 mM ABTS [2,2′-azino-bis­(3-ethylbenzothiazoline-6-sulfonic acid) diammonium salt] with 2.45 mM potassium persulfate (K_2_S_2_O_8_) in distilled water. The solution was allowed to stand in the dark at room temperature for 12 h to ensure full radical formation. The resulting ABTS^•^+^^ solution was diluted with 10% phosphate-buffered saline (PBS, pH 7.2) to yield an absorbance of 0.700 ± 0.020 at 734 nm. For each measurement, 3.9 mL of the ABTS^•^+^^ working solution was added to 0.01 g of the sample. The mixture was incubated at 25 °C, and the decrease in absorbance at 734 nm was monitored at multiple time intervals (15 min, 30 min, 45 min, 1 h, and 24 h) using a fiber-optic probe. All the experiments were conducted in triplicate. The samples were covered with aluminum foil and stored in the dark throughout the assay period to prevent photodegradation of the reactive species.^28^

*DPPH^•^ Radical Scavenging Assay* The DPPH^•^ radical solution was prepared by dissolving 2,2-diphenyl-1-picrylhydrazyl in absolute methanol to a final concentration of 0.081 mM, corresponding to an initial absorbance of 0.8621 at 517 nm. For each assay, 3.9 mL freshly prepared DPPH solution was added to 0.01 g of the sample. The mixtures were incubated at 25 °C in the dark, and the absorbance at 517 nm was measured at the same time intervals as the ABTS^•+^ assay (15 min, 30 min, 45 min, 1 h, and 24 h) using the same probe-based spectrophotometer. All the measurements were carried out in triplicate. To maintain the assay integrity, the samples were protected from light using aluminum foil and stored in the dark between readings. The percentage of radical inhibition (I) [%] was calculated using the following equation:

1 $$I=\frac{A_{0}-A_{f}}{A_{0}}\times 100\%$$

where *A*_0_ is the absorbance of the initial ABTS^•+^/(DPPH^•^) concentration, and *A*_*f*_ is the absorbance of the remaining ABTS ^•+^/(DPPH^•^) concentration in the presence of the samples.^29^

*Determination of the Antimicrobial Potential (Minimum Inhibitory Concentration)* A total of 450 mg of aqueous extract was mixed with 600 μL of Tween 20 and 54 mL of preheated Mueller–Hinton (MH) agar (40 °C), followed by homogenization at 40 °C using a rotor-stator homogenizer at 25,000 rpm to obtain uniform emulsions.

The antimicrobial activity was assessed using the broth microdilution method in 96-well plates against *Staphylococcus aureus* (MRSA, ATCC 25923) and *Escherichia coli* (ATCC 25922). Each well was filled initially with 100 μL of MH broth. Serial 2-fold dilutions of the extract emulsions were prepared across ten wells, yielding final concentrations from 37.5 to 0.07 mg/mL. Wells 11 and 12 served as the controls: the positive control contained bacteria only (10 μL of 10^8^ CFU/mL suspension), while the negative control contained extract only. The plates were incubated at 37 °C for 24 h. postincubation, and the microbial viability was assessed by adding 30 μL of a sterile 0.04% resazurin solution prepared in Milli-Q water. The plates were incubated for an additional 4 h at 37 °C. A color change, from blue to violet, indicated bacterial metabolic activity. The minimum inhibitory concentration (MIC) was defined as the lowest concentration with no visible color change.

*HPLC Analysis* A total of 100 mg of extract was dissolved in 10 mL methanol, sonicated for 10 min, and filtered through a 0.22 μm PTFE membrane. The analyses were performed in duplicate. The phenolic acids (p-coumaric, caffeic, chlorogenic, gallic) and flavonoids (rutin, epicatechin) were quantified using an Agilent 1200 HPLC system with DAD detection and a Zorbax Eclipse XDB-C18 column (150 × 4.6 mm, 5 μm). The mobile phase consisted of 0.1% trifluoroacetic acid in Milli-Q water (A) and methanol (B), with a flow rate of 1 mL/min and a 5 μL injection volume. The column temperature was maintained at 25 °C. The detection wavelengths were set at 280, 320, and 380 nm. The gradient program was: 0 min, A:B = 90:10; adjusted linearly to 10:90 by 35 min; returned to the initial conditions by 37 min. The compounds were identified by retention time and UV spectra compared to the standards and quantified using external calibration. The results are expressed in μg/mg of the extract.

##### 2.2.4.2 Dispersions

*Physical Properties* The physicochemical properties, including pH, conductivity, and turbidity, of the dispersions were analyzed, as they are essential for understanding the characteristics of the coatings. The pH was determined using a Mettler Toledo pH meter equipped with a SevenGo Duo with a 738-ISM sensor (Mettler Toledo, USA), calibrated with buffer solutions (pH 4.00, 7.00, and 10.00). The electrode was rinsed with deionized water before each measurement, and samples were stirred for uniformity. The stabilized readings were taken in triplicate, with the average recorded. The electrical conductivity was measured using a Mettler Toledo SevenCompact conductivity meter (Mettler Toledo, USA), calibrated with a KCl standard solution. To ensure consistency, the samples were maintained at a constant temperature, and the probe was rinsed between measurements. The values were recorded in μS/cm, with triplicate readings averaged. The turbidity was measured using the Velp Scientifica TB1 turbidity meter (Velp Scientifica, Italy) following the ISO 7027 Standard. The instrument was calibrated with formazin standards, and the samples were homogenized before measurement. The readings were recorded in nephelometric turbidity units (NTU), with triplicate measurements averaged for accuracy. The surface tension measurements of the dispersions were conducted using a Krüss-K12 force tensiometer (Krüss GmbH, Germany) equipped with a Wilhelmy plate method. The instrument was calibrated prior to the measurements using Milli-Q water at 25 ± 0.1 °C to ensure accuracy.

*Rheology* The rheological properties of the solutions were analyzed using an MCR 302 Rheometer (Anton Paar GmbH, Austria). A cylindrical measuring system (CC27) was used to assess viscosity over a shear rate range of 0.001 to 100 s^–1^ of the low viscous WE and YE solutions. For the macromolecular solutions, a cone-and-plate (CP50) measuring system was employed, covering a shear rate range of 1 to 1000 s^–1^. All the measurements were performed at room temperature (25 ± 1 °C) under controlled conditions.

*Zeta Potential and Particle Size Measurements* The zeta potential (ZP) and hydrodynamic diameter (HD) of the dispersions were measured using a Litesizer500 (Anton Paar GmbH, Austria) at 25 ± 1 °C. The ZP was determined via electrophoretic light scattering (ELS), reflecting the surface charge, while the HD was assessed through dynamic light scattering (DLS), analyzing the particle diffusion. The dispersions were homogenized, and, if needed, adjusted to pH 4 with acetic acid before dilution in an omega cuvette. The data were processed using the Kalliope software (Anton Paar GmbH, Austria).

*Potentiometric Titration* The total charge of the dispersions was determined using pH-potentiometric titration with a Mettler Toledo T70 two-buret system. The titrations were performed under an inert nitrogen atmosphere to prevent atmospheric interferences. The pH was adjusted from 2.5 to 11.0 using 0.1 mol L^–1^ HCl and 0.1 mol L^–1^ KOH as titrants. All the measurements were conducted in a controlled environment to ensure accuracy and reproducibility.

*Antioxidative Activity* The antioxidant activity of the dispersions (10% pullulan, 8× MIC_WE, 8× MIC_YE, pullulan +8× MIC_WE, and pullulan +8× MIC_YE) were evaluated following the procedures outlined in Section 2.2.4.1. In this case, 3.9 mL of the respective radical solution was added to 1.0 mL of each dispersion. The absorbance was measured at 734 nm for ABTS^•^+^^ and 517 nm for DPPH^•^, using a probe-based UV–Vis spectrophotometer (Cary 60, Agilent Technologies, USA) at predefined time points (15 min, 30 min, 45 min, 1 h, and 24 h). All the measurements were carried out in triplicate. The samples were covered with aluminum foil and stored in the dark between measurements to prevent light-induced degradation. The percentage of radical scavenging activity was calculated using eq 1.

##### 2.2.4.3 Cast Films

*Thickness* The thickness of the films was measured using a dial thickness gauge, F1000/30 (Käfer Messuhrenfabrik GmbH & Co. KG, Germany)

*ATR-FTIR Spectroscopy* The ATR-FTIR spectra of the free-standing films were determined with the same method as mentioned in Section 2.2.4.1.

*Surface Properties of the Cast Films* The wettability characteristics of cast films made from 10% pullulan, 10% pullulan +8× MIC_WE, and 10% pullulan +8× MIC_YE were analyzed by measuring the contact angles using an OCA 35 Optical Contact Angle Meter and SCA 20 software (version 4.1.12, DataPhysics Instruments, Filderstadt, Germany). All the measurements were conducted in triplicate at 25 °C using 3 μL droplets of ultrapure water.

The surface free energy values, calculated as the sum of the dispersive and polar components, were determined from the contact angle data using the geometric mean method, based on the Owens-Wendt equation^30^ (see eq 2). The test liquids used included water, ethylene glycol, formamide, and diiodomethane, with their respective parameters listed in the Table 2.

2 $$\gamma_{L}\left(1+\cos\theta \right)/(2\times \sqrt{\gamma_{L}^{D})}=\sqrt{\gamma_{S}^{P}}\times \sqrt{\frac{\gamma_{L}^{P}}{\gamma_{L}^{D}}}+\sqrt{\gamma_{s}^{D})}$$

**Table 2:** Surface Tension Parameters of the Testing Liquids

	Surface free energy (mN/m)			
Liquid	$\gamma_{L}^{D}$	$\gamma_{L}^{P}$	*γ_L_*	Ref.
Water	19.90	52.20	72.10	^31^
Ethylene Glycol	29.00	19.00	48.00	^32^
Formamide	39.0	19.00	58.00	^33^
Diiodomethane	49.80	1.30	51.10	^30^

where *L* and *S* refer to the liquid and solid, respectively; *γ* ^*D*^ is the dispersive component, and *γ* ^*P*^ is the polar component of the surface energy; θ is the contact angle. The *γ* ^*D*^ and *γ* ^*P*^ of the liquids are given below in Table 2.

*Optical Properties* The optical properties of the film samples were determined by measuring the percent transmittance using a Spectraflash SF600 Plus UV–vis spectrophotometer (Datacolor, Trenton, NJ, USA). Each film sample was placed directly on the side of the spectrophotometer’s magnetic cells, with an empty test cell used as a reference. The percent transmittance was measured over the wavelength range of 200–800 nm.

For color analysis, the Spectraflash SF600 was used, with the standard illuminant D65 (LAV/Spec. Incl., d/8, D65/10°). A Xenon halogen lamp served as the light source for the experiment. The CIE Lab× color values were computed using the QC 600 software, version 3.3 (Datacolor, Trenton, NJ, USA). Additionally, the CIE total color differences were determined, to evaluate the color variations using Equation 3

3 $$\Delta E^{*}=\sqrt{{\left(\Delta L^{*}\right)}^{2}+{\left(\Delta a^{*}\right)}^{2}+{\left(\Delta b^{*}\right)}^{2}}$$

where ΔE* is the total color difference; ΔL* is the difference in brightness; Δa* is the difference at the red-green axis; and Δb* is the difference at the yellow-blue axis. Pullulan films were used as the control sample.

*Scanning Electron Microscopy (SEM)* The surface morphology of the coating samples was examined using a Quanta 200 3D scanning electron microscope (FEI, Hillsboro, USA). The SEM observations were conducted under low-vacuum conditions (60 Pa) with an accelerating voltage of 10 kV, to evaluate the microstructural features of the coatings without additional conductive coating.

*X-ray Diffraction (XRD)* The crystalline structure of all the samples was analyzed by X-ray diffraction using a D5005 X-ray diffractometer (Bruker–Siemens) at room temperature. The diffraction patterns were recorded in the 2θ range of 5°–40°, with a scanning rate of 0.03°/min, employing Cu Kα radiation at an operating voltage of 30 kV and a current of 10 mA.

*Antimicrobial Susceptibility Testing* The antibacterial efficacy of the cast films was evaluated using a modified agar diffusion assay following the Diffusion Method for Antimicrobial Susceptibility Testing (Version 13.0, 2025). The bacterial suspensions were adjusted to 0.5 McFarland standard in sterile saline and spread uniformly onto MH-agar plates using an inoculating turntable to ensure confluent growth.

The samples (sterilized prior to testing) were placed aseptically onto the inoculated agar surface and allowed to diffuse for 10 min at room temperature before plate inversion. The plates were then incubated at 37 °C for 20 h, after which the zones of inhibition (ZOI) were measured to assess the antimicrobial activity. Three replicates were performed for each sample, to ensure reproducibility.

*Antioxidative Activity* The antioxidant activity of the cast films was evaluated following the procedures outlined in Section 2.2.4.1.

## 3 Results and Discussions

### 3.1 Determination of the Characteristics of Individual Components

#### 3.1.1 ATR-FTIR Spectrum

The ATR-FTIR spectra of powders of pullulan, chestnut wood extracts, and yerba mate extracts are displayed in Figure 2a. This reveals the key functional groups that could contribute to their structural and bioactive properties, making them suitable for active packaging applications. The spectrum of pullulan revealed distinct absorption bands characteristic of polysaccharides, confirming their structural identity and functional groups. A broad peak around 3400 cm^–1^ corresponds to O–H stretching vibrations, indicating the presence of hydroxyl groups involved in intermolecular and intramolecular hydrogen bonding. The C–H stretching at 2930 cm^–1^ confirms its carbohydrate backbone.^34^ The absorption band at 1640 cm^–1^ usually corresponds to the bending vibration of water molecules, indicating the presence of moisture in the sample. The fingerprint region of 1200–600 cm^–1^ exhibits multiple peaks (Figure 2b), with those at 1150 cm^–1^,1080 cm^–1^, 998 cm^–1^ and 755 cm^–1^, attributed to C–O–C stretching from α-(1→4) and α-(1→6) glycosidic linkages essential for its branched structure.^35^ The peak 845 cm^–1^ corresponds to anomeric C–H deformation vibrations, confirming α-glycosidic bonds, a key feature distinguishing pullulan from β-linked polysaccharides such as dextran.^36^ These features suggest that pullulan provides a stable matrix for bioactive compound incorporation. This interpretation is consistent with Shingel, who reported the relevance of bands at 1080 cm^–1^ and 996 cm^–1^ for glycosidic linkage type and hydrogen bonding, and with Xiao et al., who confirmed conformational ordering in the 1200–950 cm^–1^ region during film formation.^35^

**Figure 2:**
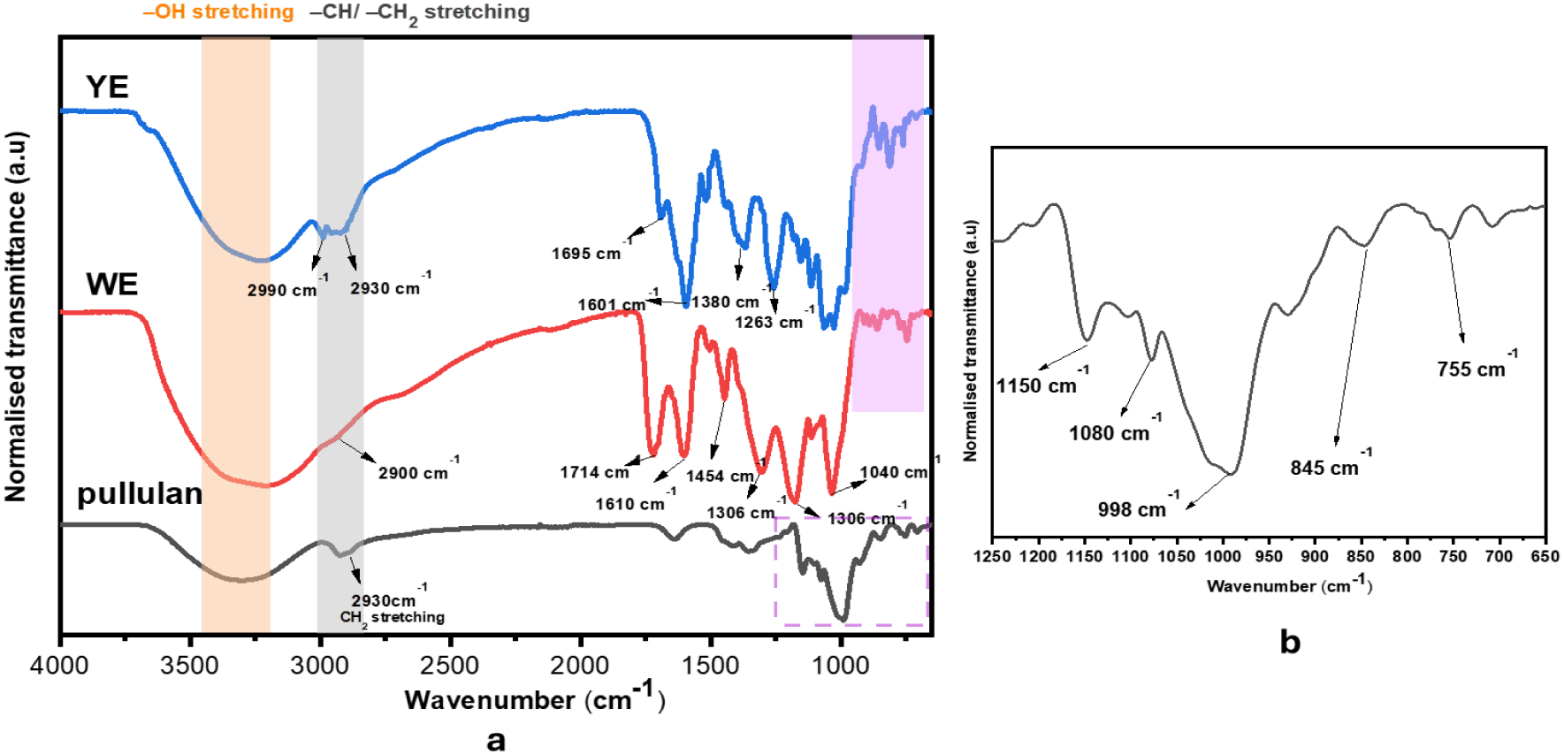
a) ATR-FTIR spectra of pullulan, wood extracts and yerba extract b) zoom-in of the fingerprint region of the pullulan spectra.

The FTIR spectrum of chestnut wood extracts (WE) revealed key functional groups associated with bioactive compounds, particularly phenolic and lignocellulosic components. A broad absorption band around 3400 cm^–1^ corresponds to O–H stretching vibrations, indicative of hydroxyl groups from phenolic compounds and carbohydrates, which could, potentially, contribute to antioxidant activity. The condensed peak at ∼2900 cm^–1^ is attributed to C–H stretching from the aliphatic chains in the lignin and other organic constituents. The presence of a distinct peak around 1714 cm^–1^ confirms the existence of C=O stretching vibrations, likely from carboxyl, ester, or conjugated ketones, which play a role in antimicrobial efficacy. This feature is consistent with Khatib et al., who reported ester and galloyl-bearing tannins from different parts of the chestnut trunk.^37,38^ The fingerprint region exhibited multiple peaks, with those at 1610 cm^–1^ and 1454 cm^–1^ corresponding to aromatic C=C stretching, which indicates lignin and tannin-derived phenolic compounds, which are known for their radical scavenging properties.^39^ The peaks at 1306 cm^–1^, 1186 cm^–1^ are associated with C–O stretching vibrations in esters, ethers, and alcohols, supporting the presence of bioactive polyphenols further. The absorption bands near 900–700 cm^–1^ suggest out-of-plane bending vibrations of aromatic rings, characteristic of the condensed tannins and flavonoids found in commonly chestnut wood extracts. The combination of these bioactive functionalities makes chestnut wood extracts a promising natural additive for sustainable and functional packaging solution. Similar functional groups were observed in the yerba mate extracts (YE), with a strong 1694 cm^–1^ C=O stretching peak attributed to esterified polyphenols and flavonoids. The pronounced peaks between 1600 and 1500 cm^–1^ suggest high aromatic content, indicative of flavonoids and condensed tannins known for their free-radical scavenging properties.^40^ The structural complexity of these extracts suggests potential synergistic effects, which will be examined further through total phenolic content (TPC), antioxidant capacity, and antibacterial studies.

#### 3.1.2 Antimicrobial Activity

The antimicrobial potential of these extracts is validated further by the Minimum Inhibitory Concentration (MIC) values presented in Table S1. The YE exhibited a uniform MIC value of 9.38 mg/mL against Gram-positive (*Staphylococcus aureus* *),* Gram-negative *(* *Escherichia coli* *)* and fungal (*Candida albicans* *)* strains, indicating a broad-spectrum antimicrobial effect. The WE demonstrated enhanced efficacy against *Staphylococcus aureus* (4.69 mg/mL), while maintaining similar inhibition levels (9.38 mg/mL) against *E. coli* and *C. albicans*. The stronger antibacterial activity of WE against *S. aureus* may be attributed to its higher concentration of tannins and phenolic aldehydes, which can disrupt the bacterial cell walls. Meanwhile, the comparable MIC values against *E. coli* and *C. albicans* suggest a shared mechanism of action between both extracts, likely linked to the phenolic hydroxyls and carbonyl functionalities interfering with the microbial metabolism.

The antimicrobial behavior of both extracts correlates with the ATR-FTIR results, confirming the phenolic and carbonyl functionalities, known to interfere with microbial membranes and metabolic pathways. These findings are consistent with previous reports highlighting phenolic-rich plant extracts as effective, natural antimicrobial agents, supporting their potential use in antimicrobial packaging to extend product shelf life.^38^

#### 3.1.3 Total Phenolic Content

The total phenolic content (TPC) analysis supports these findings further (Table S2), highlighting the bioactive potential of these extracts. The WE exhibited the highest TPC at 579.26 mg GAE/g, despite having a lower phenolic extraction yield (7.71%). This value is remarkably high when compared to the more intensive extraction protocols reported in the literature. For instance, Aimone et al. achieved TPC values of 646.36 to 802.84 mg GAE/g from chestnut wood using subcritical water extraction at elevated temperatures (100–150 °C), extended extraction times, and optimized solid-to-liquid ratios.^41^ Furthermore, the yerba mate extract (YE) contained 238.63 mg GAE/g, with a significantly higher extraction yield (29.12%), suggesting a more efficient release of phenolic compounds in aqueous media. This observation aligns with prior research on ultrarefined yerba mate, where both aqueous and methanolic extractions demonstrated substantial TPC values, 266.4 and 339.0 mg GAE/g, respectively.^42^ The pullulan showed no detectable phenolic content, reinforcing its role as a neutral polysaccharide matrix for bioactive integration rather than an active contributor to antioxidant or antimicrobial properties.

#### 3.1.4 Determination of Phenolic Compounds by HPLC

The phenolic compounds in YE and WE were identified by comparing the retention times and UV spectra with those of authenticated standards. The target compounds included phenolic acids (chlorogenic acid, caffeic acid, p-coumaric acid, gallic acid) and flavonoids (rutin, quercetin, catechin, epicatechin). The chromatograms were recorded at 280, 320, and 380 nm. The chromatogram obtained was compared with the chromatograms of the extract, and quantification is expressed in μg of analyte per mg of dry extract and is presented in the Supporting Information.

Chlorogenic acid (retention time, t_r_ = 10.17 min), caffeic acid (t_r_ = 11.25 min), p-coumaric acid (t_r_ = 14.33 min), and rutin (t_r_ = 18.83 min) were identified in the YE. Gallic acid (t_r_ = 3.80 min), epicatechin (t_r_ = 12.06 min), and ellagic acid (t_r_ = 19.74 min) were detected in the chestnut wood extract. Catechin and quercetin were not observed in either extract, while p-coumaric acid and epicatechin were only present in trace amounts. Representative chromatograms of the standard mixture and extracts are provided in the supplementary text S1.

A review of the existing literature confirms that yerba mate is a rich source of phytochemicals and antioxidant compounds, particularly phenolic acids and flavonoids. Among these, chlorogenic acid and its isomers-3,4-, 3,5-, and 4,5-dicaffeoylquinic acids are the most abundant and functionally significant. Other notable constituents include rutin, a dominant flavonoid, as well as purine alkaloids such as caffeine, theobromine, and theophylline, and various saponins. Minor quantities have also been reported of caffeic acid, p-coumaric acid, quercetin, and kaempferol. The HPLC data obtained in this study aligned with these findings.^43,44^ Chlorogenic acid was the most abundant compound in the yerba mate extract (42.11 μg/mg), followed by rutin (21.64 μg/mg). These two compounds accounted for the majority of the identified phenolics. Caffeic acid was present at 0.45 μg/mg, while p-coumaric acid appeared only in trace amounts. The observed concentration ratios agree with previous studies.^45–49^ Notably, the levels of chlorogenic acid and rutin observed here were approximately two times higher than those typically reported in the literature. For instance, the concentrations of both chlorogenic acid and rutin in this study were nearly double those reported by Deladino et al., with measured values of chlorogenic acid and rutin contents of 20.9 μg/mg and 14.7 μg/mg, respectively.^49^ This is likely because of the material-to-solvent ratio of 0.02 g/mL used in the study. In contrast, the present study employed a higher extraction ratio of 0.13 g/mL, which likely contributed to the increased extraction efficiency and compound recovery.

In the WE, ellagic acid was the dominant phenolic (42.09 μg/mg), followed by gallic acid (28.56 μg/mg), with epicatechin detected only in trace amounts. This composition aligns with the reported profiles of aqueous chestnut wood extracts, which are known to contain primarily hydrolyzable tannins and their degradation products. Such extracts typically exhibit a complex mixture of polyphenolic compounds, with gallic acid and ellagic acid as the principal components.^50^

#### 3.1.5 Antioxidative Activity

The antioxidative performance of pullulan, wood extract (WE), and yerba mate extract (YE) was evaluated using ABTS and DPPH radical scavenging assays, as presented in Figure 3. These methods provide complementary insights into the radical neutralization capacity of the samples, relevant to their potential application in active packaging systems.

**Figure 3:**
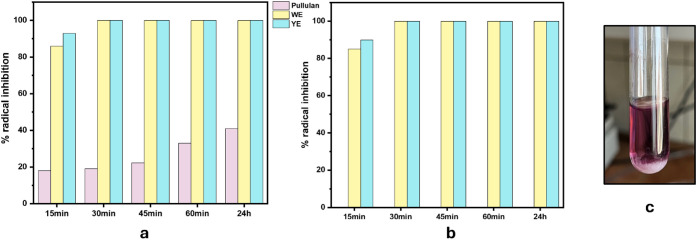
Antioxidative activity of pullulan, WE and YE a) ABTS assay, b) DPPH assay c) image of precipitate on addition of DPPH radical to pullulan -unable to measure.

In the ABTS assay (Figure 3a), YE showed the highest radical inhibition, maintaining values above 90% across all the time points. The WE also demonstrated strong activity, with inhibition exceeding 85% consistently. In contrast, the pullulan exhibited minimal radical scavenging capacity, remaining below 30% even after 1 day, which is consistent with the findings reported in the literature.^51,52^ The enhanced activity observed for YE and WE can be attributed to the presence of polyphenolic compounds and other redox-active constituents, while the low activity of pullulan is consistent with its chemical structure, which lacks the functional groups capable of effective radical scavenging, which is in accordance with the ATR-FTIR results.^22,46^

In the DPPH assay (Figure 3b), both YE and WE again exhibited strong and sustained radical inhibition, maintaining values above 85% over time. However, it was not possible to measure the antioxidative activity of pullulan using this method, due to the formation of a precipitate upon DPPH addition (Figure 3c), which interfered with the spectrophotometric measurement, nevertheless pullulan has no inherent antioxidant property. The formation of precipitate could be from solvent incompatibility and polymer destabilization. Pullulan, being water-soluble, may undergo flocculation or precipitation due to changes in solubility when exposed to methanol-based DPPH solutions. This can lead to aggregation or phase separation, especially under high-viscosity conditions, such as with the 10% (w/v) pullulan solution used here. Minor solvent disturbances under these conditions can result in localized crowding, promoting gelation or precipitation, and rendering the system unsuitable for accurate optical measurement.^7,53^

Both the ABTS and DPPH assays rely on different underlying reaction mechanisms. ABTS measures primarily single electron transfer (SET), while DPPH can involve both SET and hydrogen atom transfer (HAT) mechanisms.^54^ Thus, the high radical inhibition values observed for YE and WE in both the ABTS and DPPH assays, suggest the presence of antioxidants with multiple radical scavenging mechanisms; capable of engaging in both electron transfer and hydrogen atom transfer mechanisms. The ability of plant extracts to perform well in both assays indicates their capacity to scavenge free radicals through multiple pathways, enhancing their overall antioxidant efficacy.^55,56^

These findings highlight the chemical versatility of the extracts and support their suitability for incorporation into biopolymer matrices where antioxidative protection is required.^57^ Importantly, the antioxidant performance correlates well with the phenolic composition and total phenolic content determined by HPLC and TPC analyses, particularly the high abundance of chlorogenic acid and rutin in YE and ellagic and gallic acids in WE. Pullulan itself is not antioxidant, but it is a highly advantageous biopolymer which is neutral, water-soluble, and known for its excellent film-forming ability, oxygen barrier performance, edibility, biodegradability, and microbial origin. Its compatibility with industrial coating processes and suitability for direct food contact make it an ideal carrier. Therefore, combining pullulan with bioactive plant extracts is a well-founded strategy, to endow the coating with active antioxidant functionality while maintaining its sustainable and food-safe profile.^58^ Overall, these results confirm that, while pullulan alone cannot offer antioxidative activity, it can be enhanced significantly through the integration of plant-based extracts such as YE and WE. This strategy is particularly advantageous for the development of active packaging materials aimed at extending product shelf life and mitigating oxidative degradation.

#### 3.1.6 Correlation between Total Phenolic Content, Phenolic Profile, and Antioxidant Activity

The combined results of the TPC analysis, antioxidant assays, and HPLC profiling reveal a clear structure–activity relationship governing the antioxidative performance of the investigated extracts. The high radical scavenging activity observed for both YE and WE correlate strongly with their elevated total phenolic content, confirming that phenolic compounds are the primary contributors to antioxidant functionality. However, the results also demonstrate that antioxidant efficiency is not determined solely by the total phenolic content but is strongly influenced by the qualitative phenolic composition.

The chestnut wood extract exhibited the highest TPC, which is consistent with its pronounced antioxidant activity in both ABTS and DPPH assays. HPLC analysis revealed ellagic acid and gallic acid as the dominant phenolics in WE, compounds known for their strong electron-donating capacity and ability to stabilize free radicals through resonance-stabilized phenoxyl structures. These hydrolyzable tannin derivatives are particularly effective in single electron transfer mechanisms, which explain the high and sustained radical inhibition observed experimentally.

In contrast, yerba mate extract displayed a lower overall TPC, yet achieved comparable or even superior radical scavenging efficiency. This behavior can be attributed to its distinct phenolic profile, dominated by chlorogenic acid and rutin. Chlorogenic acid, containing multiple hydroxyl groups and conjugated aromatic systems, is highly effective in both electron transfer and hydrogen atom transfer mechanisms, while rutin contributes additional radical stabilization and metal-chelating effects. The synergistic presence of these compounds likely compensates for the lower total phenolic concentration, resulting in strong antioxidant performance across both assays.

Pullulan, lacking detectable phenolic compounds and functional antioxidant groups, exhibited negligible radical scavenging activity, confirming that its role within the system is purely that of a neutral carrier matrix. Importantly, the preservation of high antioxidant activity upon incorporation of YE and WE demonstrate that the bioactive functionality is retained and can be effectively translated into pullulan-based formulations.

Overall, these findings highlight that both the quantity and molecular nature of phenolic compounds govern antioxidant performance and underscore the importance of coupling TPC measurements with detailed HPLC profiling when designing and evaluating bioactive coating systems. This integrated understanding is essential for the rational development of functional biopolymer coatings with predictable antioxidative performance.

### 3.2 Determination of the Characteristics of the Dispersion

#### 3.2.1 Physicochemical Characteristics

The physicochemical properties of the formulated colloidal solutions-dispesrions (Figure 4), including pH, electrical conductivity, turbidity, and surface tension, were evaluated as indicators of stability and applicability for pullulan-based coating systems.

**Figure 4:**
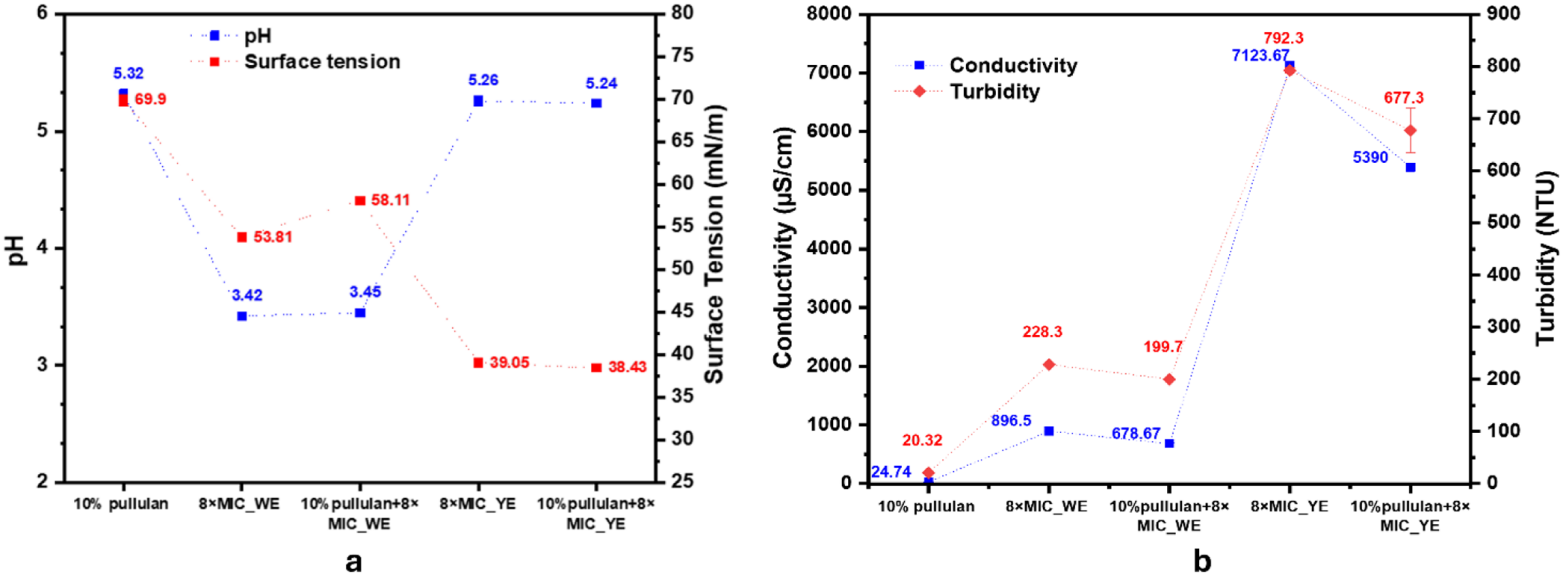
Physicochemical properties of the formulated solutions (a) pH and surface tension (b) conductivity and turbidity.

The 10% pullulan solution exhibited a slightly acidic pH (5.35 ± 0.02). The incorporation of 8× MIC_WE reduced the pH significantly to ∼3.45, confirming the acidic nature of WE, while YE had a negligible effect, maintaining the pH close to that of neat pullulan. These results indicate that WE influences the solution acidity strongly, whereas YE preserves the native pH of the pullulan matrix

The electrical conductivity increased markedly upon extract addition. The neat pullulan showed low conductivity (24.74 ± 0.85 μS/cm), while the WE- and YE-containing formulations reached 678.67 ± 12.95 μS/cm and 5390 ± 40.63 μS/cm, respectively. The higher conductivity of the YE-based systems reflects a greater abundance of ionic species such as peptides, amino acids, and nucleotides, as well as inorganic ions like calcium, magnesium, potassium, phosphates, etc., which may enhance the electrostatic interactions and polymer chain mobility within the coating matrix.^59^

The turbidity increased significantly with extract incorporation, rising from 20.32 ± 1.89 NTU for pullulan to 199.7 ± 0.57 NTU (WE) and 677.3 ± 43.02 NTU (YE). This increase suggests the presence of dispersed colloidal components and enhanced molecular interactions, which may contribute to thicker coatings, although with reduced transparency.^60^

The surface tension decreased substantially upon extract addition. While neat pullulan exhibited high surface tension (69.9 ± 0.68 mN/m), the WE and YE reduced it to 58.11 ± 0.12 mN/m and 38.43 ± 0.17 mN/m, respectively. The pronounced surface tension reduction with YE indicates the presence of surface-active components, may improving the wetting and spreading behavior, critical for achieving uniform coatings on polymeric substrates. Importantly, the reduced surface tension of the dispersions facilitates coating application on low-surface-energy and relatively inert packaging polymers such as PLA, PET, and polypropylene (PP), thereby improving wettability and, consequently, coating adhesion and coverage homogeneity.

Overall, WE and YE modify the physicochemical profile of pullulan solutions distinctly. WE influence primarily acidity, while YE enhances the conductivity and interfacial properties strongly. These changes suggest improved wettability, spreading, and potential coating adhesion, supporting the suitability of extract-modified pullulan systems for functional food-packaging coatings.^61^

#### 3.2.2 Rheological Behavior

The rheological profiles of the pullulan-based formulations and extracts were evaluated to assess their suitability for coating applications. Table 3 presents the intrinsic viscosities (η_a_) of the individual and blended systems, while Figure 5 illustrates the flow behavior across a range of shear rates. These data provide complementary insights into the structure and interactions within the pullulan-based dispersions.

**Figure 5:**
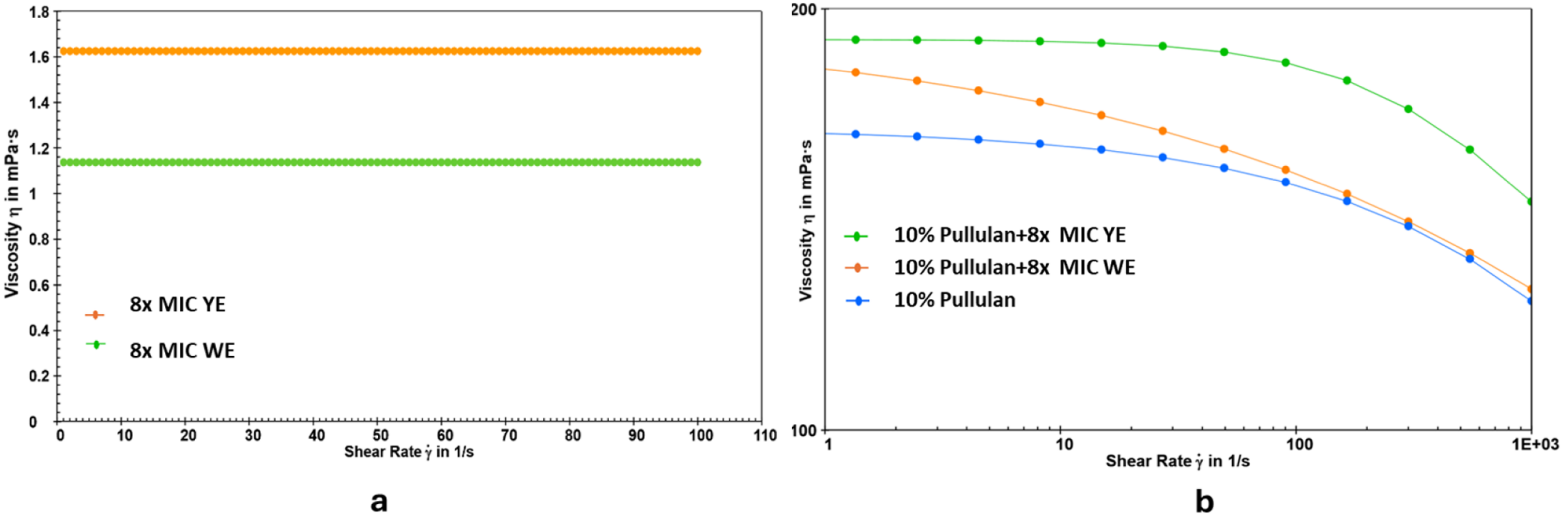
Rheological properties of the dispersions a) standalone extract b) pullulan and extracts in combination with pullulan.

**Table 3:** Intrinsic Viscosities of the Dispersions

Sample	Intrinsic viscosity, *η_α_* mPa/s
0% Pullulan	164.43
10% Pullulan+ 8× MIC_YE	194.4
10% Pullulan+ 8× MIC_WE	190.18
8× MIC_YE	1.62
8× MIC_WE	1.13

The neat 10% pullulan exhibited a viscosity of 164.43 mPa·s. The incorporation of bioactive extracts at 8× MIC increased the viscosity to 194.40 mPa·s for YE and 190.18 mPa·s for WE, indicating enhanced intermolecular interactions within the pullulan matrix. This increase is attributed to hydrogen bonding between the pullulan hydroxyl groups and phenolic compounds in the extracts, consistent with the FTIR observations and previous reports on phenolic-rich systems. In contrast, the standalone YE and WE solutions exhibited very low viscosities (1.62 and 1.13 mPa·s, respectively), confirming that viscosity enhancement arises from polymer–extract interactions rather than the extracts alone. All the pullulan-based formulations displayed shear-thinning behavior, with viscosity decreasing as the shear rate increased. This pseudoplastic response is advantageous for coating processes, enabling easy spreading during application while maintaining structural integrity at rest. The extract-only solutions showed Newtonian behavior, confirming the absence of significant molecular entanglement in the absence of the polymer matrix further.^62^

Overall, the combination of increased intrinsic viscosity and shear-thinning behavior indicates that extract-modified pullulan formulations offer favorable processability and film-forming potential. These rheological characteristics support their applicability as functional coating systems, while the low-viscosity extract solutions may also be suitable as secondary layers for bioactive delivery in multilayer coating designs.

The flow behavior, depicted in the viscosity versus shear rate plots (Figure 5), supports these findings further. All the pullulan-based systems exhibited shear-thinning (pseudoplastic) behavior, characterized by a decrease in viscosity with the increasing shear rate. This is beneficial for coating applications, as it facilitates smooth spreading and uniform film formation during application processes such as brushing, rolling, or spraying. The shear-thinning nature also reflects internal structural changes, where polymer chains align and disentangle under shear.^63^

In contrast, the neat 8× MIC_YE and 8× MIC_WE dispersions demonstrated Newtonian behavior, maintaining constant viscosities across all the shear rates. This lack of shear dependence confirms the absence of significant molecular entanglement or interaction in the extract-only systems, further supporting the notion that viscosity enhancements in the composite systems result from synergistic polymer–extract interactions. These rheological characteristics have important implications for formulation design. Systems exhibiting moderate shear-thinning and elevated intrinsic viscosity offer improved processing stability and end-use performance in applications such as edible films, coatings, and encapsulation matrices, where flow behavior governs the spreadability, uniformity, and mechanical strength. The observed decrease in viscosity at higher shear rates ensures good processability and ease of application.^64,65^ Such an approach could be particularly valuable in active packaging applications, where prolonged protection is desired against microbial contamination and oxidative degradation. Further studies focusing on adhesion, drying behavior, and release kinetics will be essential, to validate the performance of this multilayer coating system.^66,67^

#### 3.2.3 Zeta Potential Measurements

The surface charge characteristics of the developed formulations serve as a critical indicator of colloidal stability and was assessed via zeta potential measurements across a pH range of 2–10 (Figure 6).

**Figure 6:**
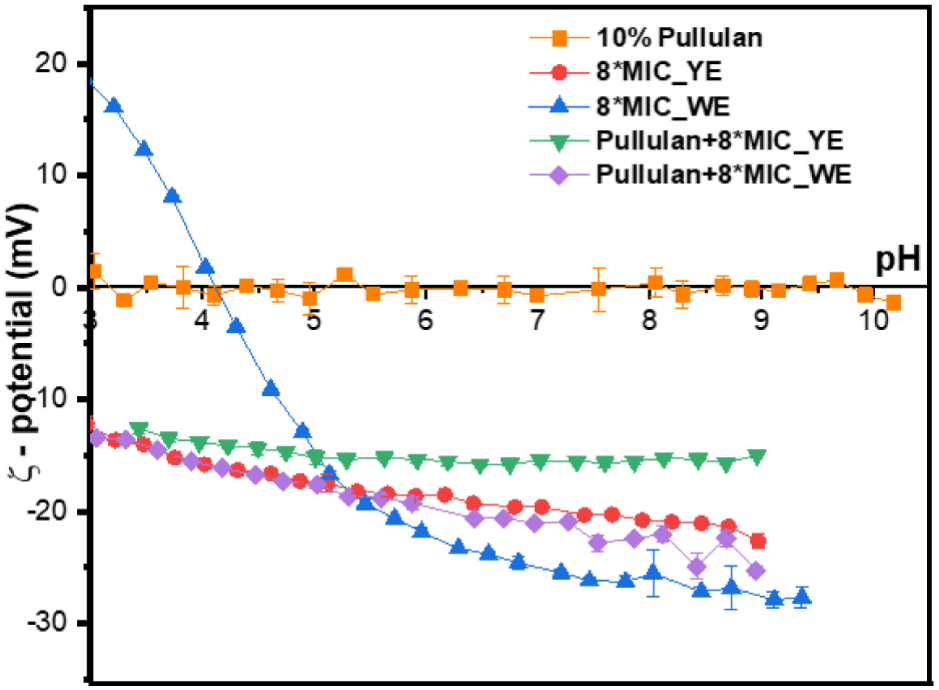
Zeta potential across pH of the dispersions.

The neat 10% pullulan exhibited near-neutral zeta potential values (∼0 mV) across the entire pH range, reflecting its nonionic polysaccharide structure and limited intrinsic colloidal stability. This behavior is consistent with the previously reported data for pure pullulan systems.^68^

In contrast, the 8× MIC_WE and 8× MIC_YE dispersions showed increasingly negative zeta potential values with rising pH, indicating the presence of ionizable acidic groups derived from the phenolic and carboxylic constituents. The WE extract exhibited the most pronounced shift, decreasing from approximately +20 mV at pH 2 to −28 mV at pH 7, followed by a plateau at higher pH. This behavior is attributed to the higher content of readily dissociable phenolics, such as gallic acid and epicatechin,^69^ whereas the phenolic profile of YE contains less easily dissociable compounds, like chlorogenic acid, caffeic acid, p-coumaric acid, and rutin, because of their structural features like fewer adjacent hydroxyls, the presence of glycosylation or esterification, and lack of strong resonance or electron-withdrawing effects that would otherwise stabilize the phenoxide ion and promote dissociation, supported by Rice-Evan. The increased negative surface charge is associated widely with improved colloidal stability and has been reported for other phenolic-rich plant extracts.^70^

The pullulan—extract formulations exhibited intermediate zeta potential profiles between those of neat pullulan and the corresponding extracts. The Pullulan +8× MIC_YE maintained relatively stable values around −12 mV across the pH range, while Pullulan +8× MIC_WE reached approximately −25 mV at neutral to alkaline pH. This behavior suggests effective incorporation of the extracts within the pullulan matrix, with charge attenuation likely arising from hydrogen bonding and weak electrostatic interactions between the polysaccharide chains and phenolic groups. The more pronounced negative shift observed for WE-based formulations is consistent with the higher content of deprotonable phenolic acids, particularly ellagic and gallic acids. This significant shift indicates extensive deprotonation of the phenolic acids, which has been observed similarly in polyphenol-rich systems such as tea catechin and seaweed-derived extracts.^71,72^

Overall, the increased negative zeta potential at neutral pH indicates enhanced dispersion stability, reducing the likelihood of particle aggregation. Such stability is particularly critical for coating formulations, as it ensures homogeneous distribution of bioactive components, prevents phase separation during storage, and enables reproducible wetting and film formation during application. Consequently, the observed zeta potential behavior supports the suitability of these formulations for active packaging applications requiring stable, shelf-ready colloidal systems with long-term functional performance.^73,74^

#### 3.2.4 Particle Size Measurements

The particle size measurements were performed at pH 7, where the formulations exhibited maximum colloidal stability based on the zeta potential analysis, ensuring representative and application-relevant dispersion characteristics, to evaluate the dispersion behavior and structural organization of the formulated coatings. The hydrodynamic diameter (HD) and polydispersity index (PDI) of the systems are summarized in Figure 7.

**Figure 7:**
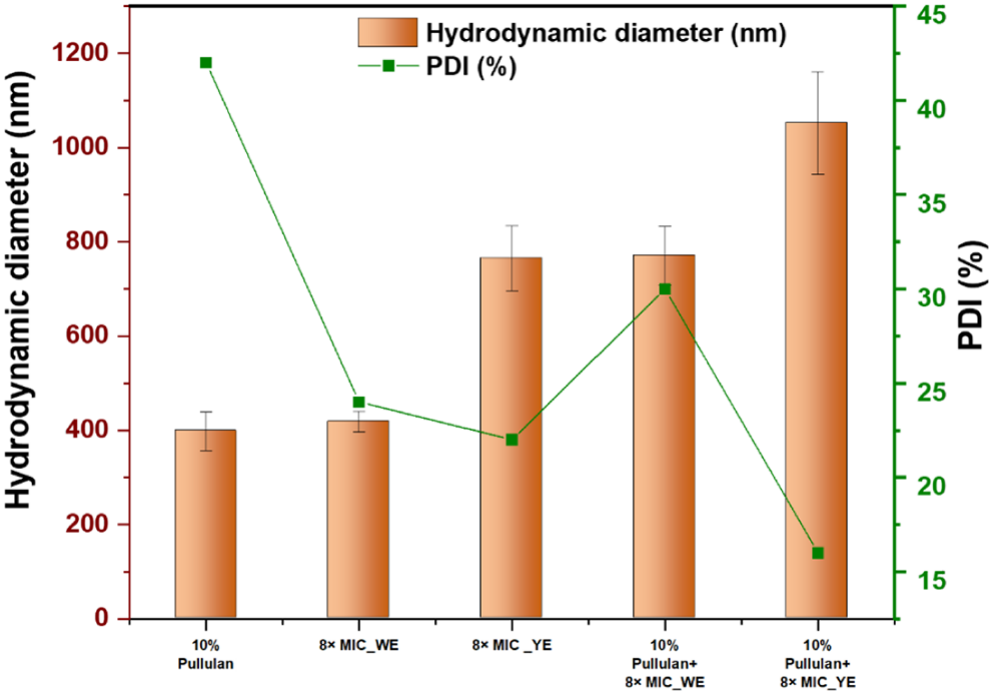
Hydrodynamic diameter and Poly dispersity index (PDI) of the dispersions.

The neat 10% pullulan exhibited an average HD of 397.57 ± 40.91 nm with the highest PDI (42%), indicating a broad and heterogeneous particle size distribution. This behavior is typical of flexible polysaccharide chains undergoing varying degrees of entanglement and aggregation in aqueous media. Despite its high polydispersity, the relatively small particle size supports its suitability for uniform film formation.^75^

The standalone extract solutions displayed larger particle sizes, with HD values of 765.06 ± 69.32 nm for 8× MIC_YE and 418 ± 21.70 nm for 8× MIC_WE. The substantially higher YE value indicates a greater degree of self-association, likely arising from strong hydrogen bonding and π–π stacking interactions among the polyphenolic compounds, consistent with previously reported interaction mechanisms.^76,77^ Both the 8× MIC_YE and 8× MIC_WE solutions had lower PDIs (22% and 24%, respectively), indicating more uniform particle populations relative to pullulan. This combination of relatively large particle size and low dispersity suggests the formation of well-defined supramolecular assemblies rather than uncontrolled aggregation.

Incorporation of the extracts into the pullulan matrix led to pronounced changes in particle size and dispersity. The Pullulan +8× MIC_WE formulation exhibited an increased HD of 770.9 ± 61.81 nm with a reduced PDI (30.79%), indicating the formation of moderately uniform aggregates through polymer–polyphenol interactions. Notably, the Pullulan +8× MIC_YE showed the largest particle size (1052 ± 109 nm) while exhibiting the lowest PDI (16%), suggesting the formation of large, yet highly uniform and stable colloidal assemblies stabilized by strong polysaccharide–polyphenol interactions. Such behavior of large size and low dispersity combination implies the formation of stable, highly uniform colloidal assemblies, likely stabilized by strong and specific interactions between the pullulan and polyphenolic components from the YE, supporting the observations from N’Guessan et al. on polysaccharide-polyphenol complex stability.^78^

These results are consistent with the zeta potential data, where the extract-containing pullulan systems—particularly Pullulan +8× MIC_YE, exhibited higher negative surface charge, counteracting aggregation despite the increased particle size. The increase in particle dimensions also correlates with the enhanced intrinsic viscosity observed in the rheological measurements, reflecting greater molecular entanglement and network formation. From a coating perspective, such colloidal architectures are advantageous, as they promote formulation stability while enabling controlled flow, uniform spreading, and reproducible film formation during surface applications. Together, these findings highlight the importance of controlling the particle size and polydispersity to achieve stable, homogeneous dispersions with favorable rheological behavior, which is essential for the performance of active packaging coatings.

#### 3.2.5 Potentiometric Titration

The charge per mass (Q/m) profile provides complementary insight into the charging behavior, acid–base characteristics, and colloidal stability of the dispersions, as shown in Figure 8. The potentiometric titration results support the zeta potential findings strongly and validate the acid–base behavior anticipated from the HPLC-identified phenolic composition of WE and YE experimentally.

**Figure 8:**
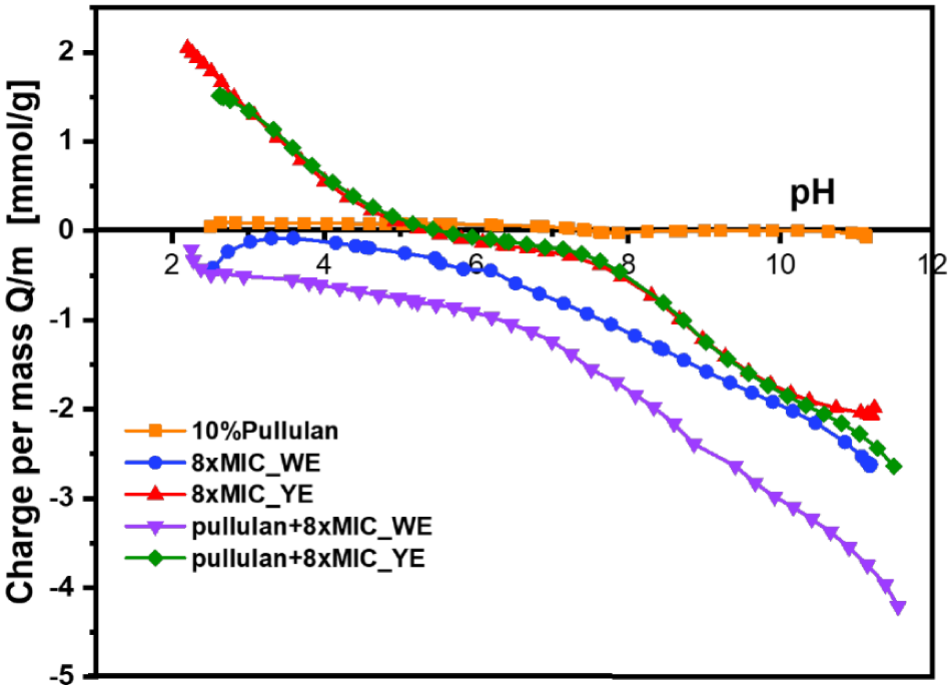
Potentiometric titration curves.

As expected, the neat pullulan exhibited negligible buffering capacity across the pH range of 2–10, reflecting its neutral polysaccharide structure and lack of ionizable functional groups. This behavior is consistent with its linear α-(1→4)/(1→6)-linked glucan backbone. In contrast, both the 8× MIC_WE and 8× MIC_YE displayed pronounced titratable acidity, indicating a high density of deprotonatable groups, likely attributed to phenolic acids, flavonoids, and organic acids, as reported previously for plant-derived polyphenolic extracts.^69^

The 8× MIC_WE formulation exhibited broad, polyprotic buffering behavior between pH 3 and 6, attributable to the ellagic and gallic acids containing multiple phenolic hydroxyl groups with overlapping p*K*a values. This gradual titration profile is characteristic of hydrolyzable tannin-rich wood extracts, where sequential deprotonation occurs across a wide pH range. In comparison, the 8× MIC_YE showed a two-stage buffering response: low-pH dissociation (pH 2.5–4.5) associated with the chlorogenic and caffeic acids, followed by higher-pH deprotonation (pH 8–10) of the catechol groups from flavonoids such as rutin, consistent with prior studies on yerba mate phenolics.^45,59^

Upon incorporation into the pullulan matrix, both extracts governed the overall charging behavior of the dispersions, yielding intermediate titration and zeta potential profiles. The minimal electrostatic contribution from pullulan indicates that polymer-extract interactions are dominated by noncovalent forces, primarily, hydrogen bonding and hydrophobic interactions between the hydroxyl-rich pullulan chains and aromatic phenolic moieties. In the Pullulan +8× MIC_WE formulation, the attenuated, yet persistent polyprotic buffering suggests partial engagement of the ellagic and gallic acid hydroxyl groups through hydrogen bonding, reducing the number of freely titratable protons. Similarly, the Pullulan +8× MIC_YE retained a titration profile comparable to the neat extract, indicating that the carboxyl groups remained largely unbound, while the flavonoid hydroxyls participated in the polysaccharide interactions. This interpretation aligns with prior studies demonstrating the ability of pullulan to form associative networks with phenolic compounds nonionic mechanisms.^70^

Overall, these results confirm that incorporation of phenolic-rich extracts into a neutral pullulan matrix produces stable, anionic colloidal systems. The close agreement between the Q/m titration profiles and the ζ-potential trends indicates that the progressive deprotonation of phenolic functional groups directly governs the surface charge development of the dispersions across the pH range. The combined potentiometric titration and zeta potential analyses demonstrate that noncovalent polysaccharide–polyphenol interactions govern dispersion stability, while preserving the ionizable groups essential for functionality. Specifically, the pH regions associated with increased charge density in the Q/m curves correspond directly to the more negative ζ-potential values, confirming a strong correlation between bulk acid–base behavior and interfacial electrokinetic properties.

#### 3.2.6 Antioxidative Activity

The antioxidative performance of the formulated dispersions was evaluated using ABTS and DPPH radical scavenging assays (Figure 9a,b), which provide complementary insight into their free-radical neutralization capacity relevant for active packaging applications.

**Figure 9:**
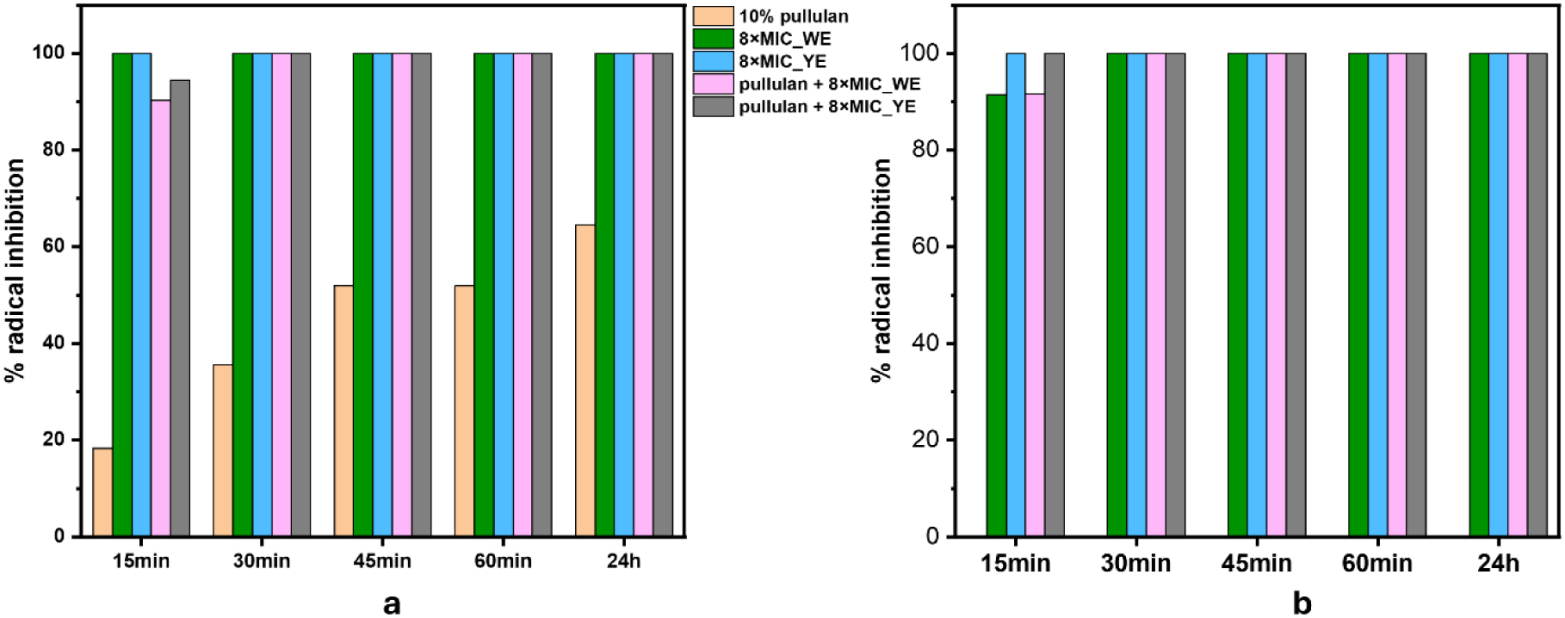
Antioxidative activity of the solutions/dispersions a) ABTS assay results b) DPPH assay results.

In the ABTS assay (Figure 9a), both the standalone extract formulations, (8× MIC_WE and 8× MIC_YE), exhibited rapid and sustained radical inhibition exceeding 95% throughout the 24 h measurement period. The corresponding pullulan-based formulations (Pullulan +8× MIC_WE and Pullulan +8× MIC_YE) showed comparable inhibition levels, indicating that incorporation into the pullulan matrix did not compromise the antioxidant activity. In contrast, the neat 10% pullulan displayed limited scavenging ability (<40%), consistent with the absence of redox-active functional groups in its structure.

The DPPH assay results (Figure 9b) reinforce these observations. Both extract-only formulations and their respective pullulan-integrated counterparts maintained near-complete radical inhibition (≥95%) across all time points. However, as with the individual extract solutions, the 10% pullulan sample was excluded from the DPPH measurements due to visible precipitation following the DPPH addition, which interfered with the optical quantification (as previously shown in Figure 3c). The observed precipitation was likely due to solvent incompatibility and viscosity-induced destabilization, wherein the methanol-based DPPH reagent induces the conformational collapse or aggregation of the water-soluble pullulan polymer.^53^

The comparable antioxidant performance of the extract-only and pullulan-based systems confirms that phenolic compounds remain chemically accessible and active after incorporation into the polymer matrix, with minimal sequestration or deactivation. Moreover, the sustained radical scavenging over 24 h highlights the potential of these formulations to provide prolonged oxidative protection. This behavior is in good agreement with the high total phenolic content determined for both extracts and with the HPLC-identified phenolic profiles dominated by chlorogenic acid and rutin in YE, and ellagic and gallic acids in WE. These individual compounds are well-known, in their pure form, to exhibit strong ABTS and DPPH radical scavenging activity through single-electron transfer and hydrogen atom transfer mechanisms.

Thus, the antioxidant performance of the formulated dispersions reflects not only the overall phenolic concentration, but also the presence of highly efficient phenolic species, whose activity is preserved upon formulation.

Importantly, the high and sustained radical scavenging activity observed in the liquid dispersions indicates that the antioxidants are not kinetically trapped within the polymer network, but remain sufficiently mobile and accessible, a prerequisite for effective antioxidant functionality after film formation and during interfacial contact with food or the surrounding atmosphere.

### 3.3 Determination of the Cast Film Characteristics

To understand the applicability of pullulan and its mixtures with plant extracts as functional coatings for packaging materials better, we hypothesized that these formulations adhere to surfaces in the form of continuous films. Therefore, we prepared freestanding films using neat pullulan and its combinations with bioactive extracts, incorporating glycerol as a plasticizer to enhance film flexibility. Although cast films do not fully replicate industrial coating processes, they serve as a well-established and controlled model system for elucidating structure–property relationships relevant to surface-applied coatings. Film formation is a critical prerequisite for coating performance, as it affects surface coverage, adhesion, mechanical integrity, and barrier properties directly. In addition to the functional evaluations such as antimicrobial activity, UV-shielding, optical transparency, and oxygen permeability, scanning electron microscopy (SEM) and X-ray diffraction (XRD) were employed to investigate the film̀s surface morphology, structural homogeneity, and changes in polymer chain organization induced by extract incorporation. These solid-state analyses enable direct correlation between the molecular and colloidal interactions identified in the liquid formulations and the macroscopic performance of the resulting films. Together, these analyses bridge the gap between dispersion-level characterization and solid-state film performance, providing a comprehensive assessment of the suitability of these systems as biobased coatings for food-packaging applications.

#### 3.3.1 Thickness

The measured thickness of the cast films is displayed in Figure 10. The neat pullulan film had the lowest thickness (0.105 ± 0.007 mm), while the films formed from pullulan +8× MIC_WE and pullulan +8× MIC_YE displayed increased thicknesses of 0.162 ± 0.015 mm and 0.215 ± 0.003 mm, respectively. This trend can be correlated to changes in the hydrodynamic diameter (HD) and intrinsic viscosity of the solutions. The observed increase in thickness across the formulations corresponds closely with a rise in the intrinsic viscosity and hydrodynamic diameter, particularly in the pullulan +8× MIC_YE system, which exhibited the largest particle size (1052 ± 109 nm). The formation of larger colloidal structures likely enhances intermolecular interactions and entanglement, contributing to greater resistance to flow, and, consequently, higher viscosity. This combination of increased size and viscosity is reflected in the resulting film morphology, where reduced mobility during casting leads to the formation of thicker and more cohesive films producing the thickest film (0.215 mm). In this context, film thickness can be viewed as a macroscopic manifestation of the underlying colloidal architecture and rheological behavior of casting dispersions. A similar trend was observed in the pullulan +8× MIC_WE formulation, which also showed elevated particle size and viscosity, ultimately producing a thicker film than the control pullulan film. From a coating perspective, this thickness modulation through formulation-level control is advantageous, as it enables tuning of film coverage and barrier performance without altering the polymer concentration or processing conditions.

**Figure 10:**
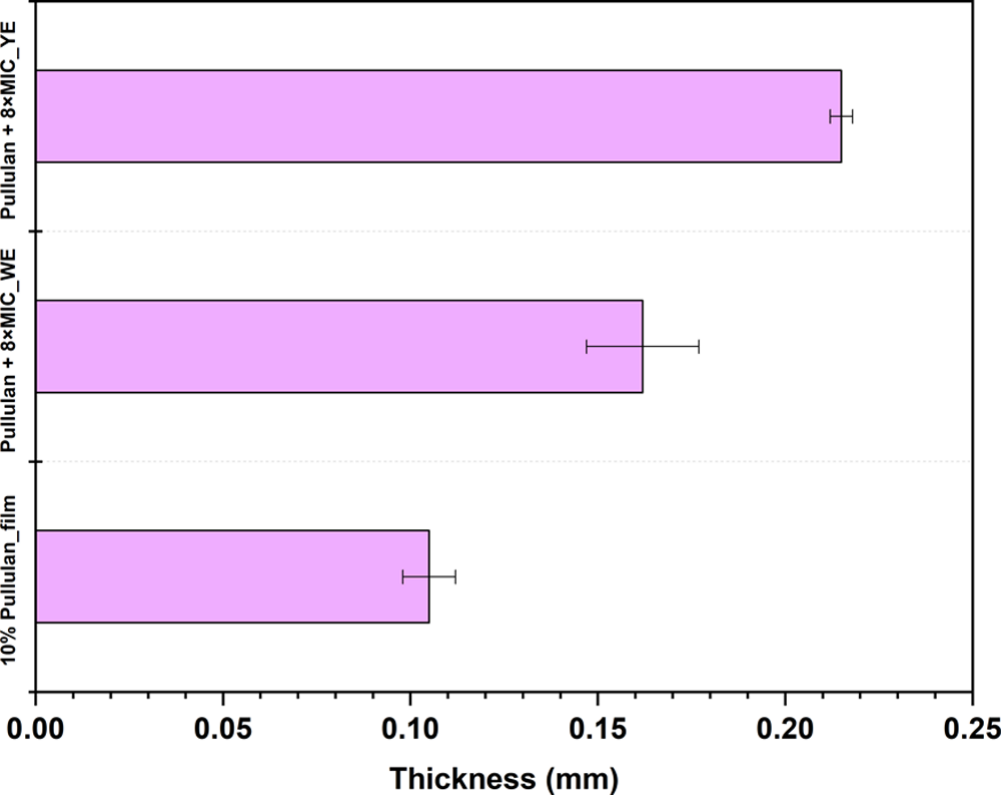
Thickness of the cast films.

#### 3.3.2 ATR-FTIR

ATR-FTIR spectroscopy was employed to elucidate the molecular interactions on the surface and confirm the incorporation of bioactive extracts onto the pullulan-based film matrices. The ATR-FTIR spectra of 10% pullulan films and films incorporated with 8× MIC_WE and 8× MIC_YE along with glycerol as a plasticizer, revealed distinct spectral changes due to molecular interactions between the pullulan matrix and the extract components, as displayed in Figure 11.

**Figure 11:**
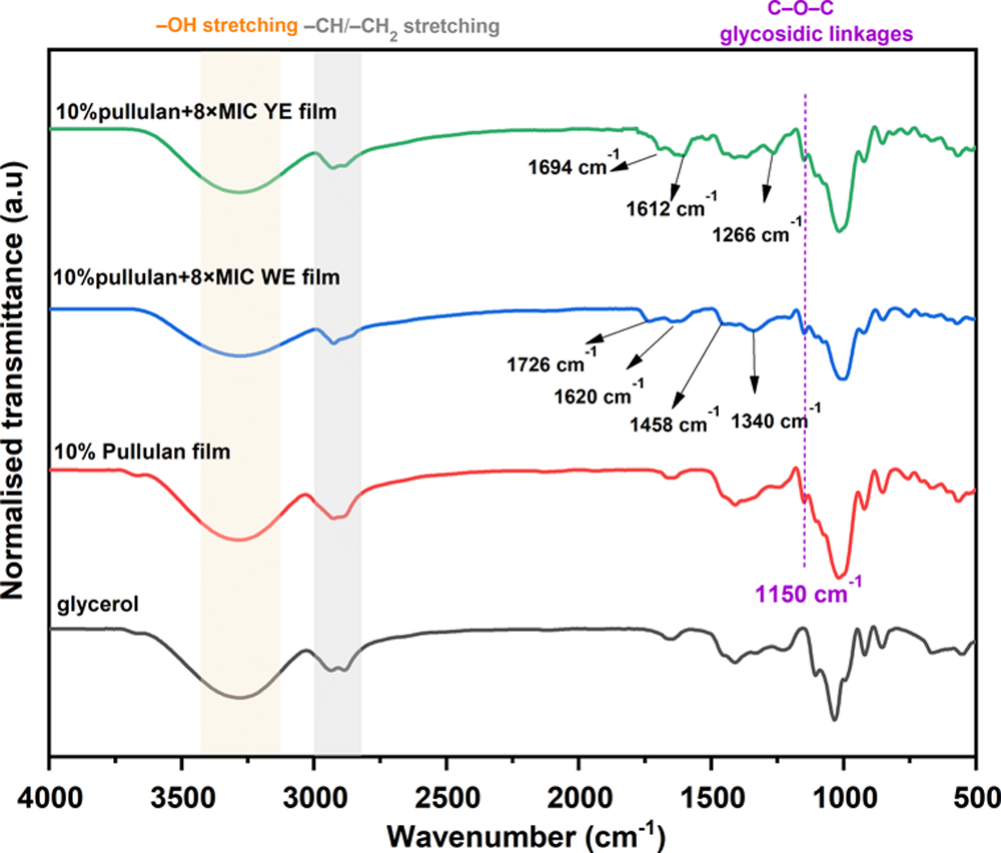
ATR-FTIR spectra of the films and glycerol.

All the samples exhibited a broad absorption band in the range of 3300–3500 cm^–1^, corresponding to the O–H stretching vibrations. This feature indicates extensive hydrogen bonding between the hydroxyl groups. The consistency of the intensity and shape of this band across the pure pullulan, extract-loaded films, and the glycerol reference, indicates that the addition of the extracts does not disrupt the primary polysaccharide network. Also, the similarity of this spectral region to glycerol, known for its hydrogen bonding ability, confirms its role as a plasticizer that facilitates interchain interactions. The absorbances in the 2900–3000 cm^–1^ range are assigned to the symmetric and asymmetric stretching vibrations of the CH_2_ and CH groups. These peaks occurred consistently in all the samples, with no significant variations in intensity or position, indicating that the hydrocarbon backbone of pullulan and the glycerol additive remains structurally stable regardless of the presence of YE or WE.

Additional spectral features associated with the extracts were retained in the composite films, although with slight shifts or broadening. In the pullulan +8× MIC_YE films, bands corresponding to C=O stretching (∼1695 cm^–1^) and aromatic C=C vibrations (∼1601 cm^–1^) were observed at ∼1694 cm^–1^ and ∼1612 cm^–1^, respectively, indicating the successful incorporation of polyphenolic components. Similarly, the phenolic C–O and C–H bending vibrations appearing at 1380 and 1263 cm^–1^ in the neat YE were present as broadened shoulders in the composite films, reflecting the redistribution of interactions within the polymer matrix. The pullulan +8× MIC_WE films showed analogous behavior, with a broadened band around ∼1700 cm^–1^ and shoulders at ∼1726 and ∼1620 cm^–1^, consistent with the presence of aromatic and carbonyl-containing phenolics from the WE. Overall, the ATR-FTIR results confirmed the successful functionalization of pullulan films with bioactive extracts through noncovalent interactions, predominantly hydrogen bonding. The observed spectral features and band broadening trends are consistent with the noncovalent polysaccharide–polyphenol interactions previously identified in the colloidal dispersions, indicating that the interaction mechanisms established at the formulation level are preserved upon film formation. The assignment of aromatic C=C and carbonyl stretching bands is in agreement with the phenolic acids and flavonoids identified by HPLC, whose characteristic functional groups are known to participate in hydrogen bonding interactions with hydroxyl-rich polysaccharides. Moreover, the preservation of characteristic pullulan bands alongside the extract-specific features indicates that the bioactive compounds remained accessible at the film surface of the packaging material, supporting the enhanced physicochemical and biofunctional properties required for active food-packaging applications.

#### 3.3.3 Wettability and Surface Free Energy

The contact angle and surface free energy (SFE) measurements provided insight into the wettability and interfacial behavior of the cast films, which are critical parameters for their application as film forming coatings. Figure 12 presents the measured surface properties of the prepared cast films.

**Figure 12:**
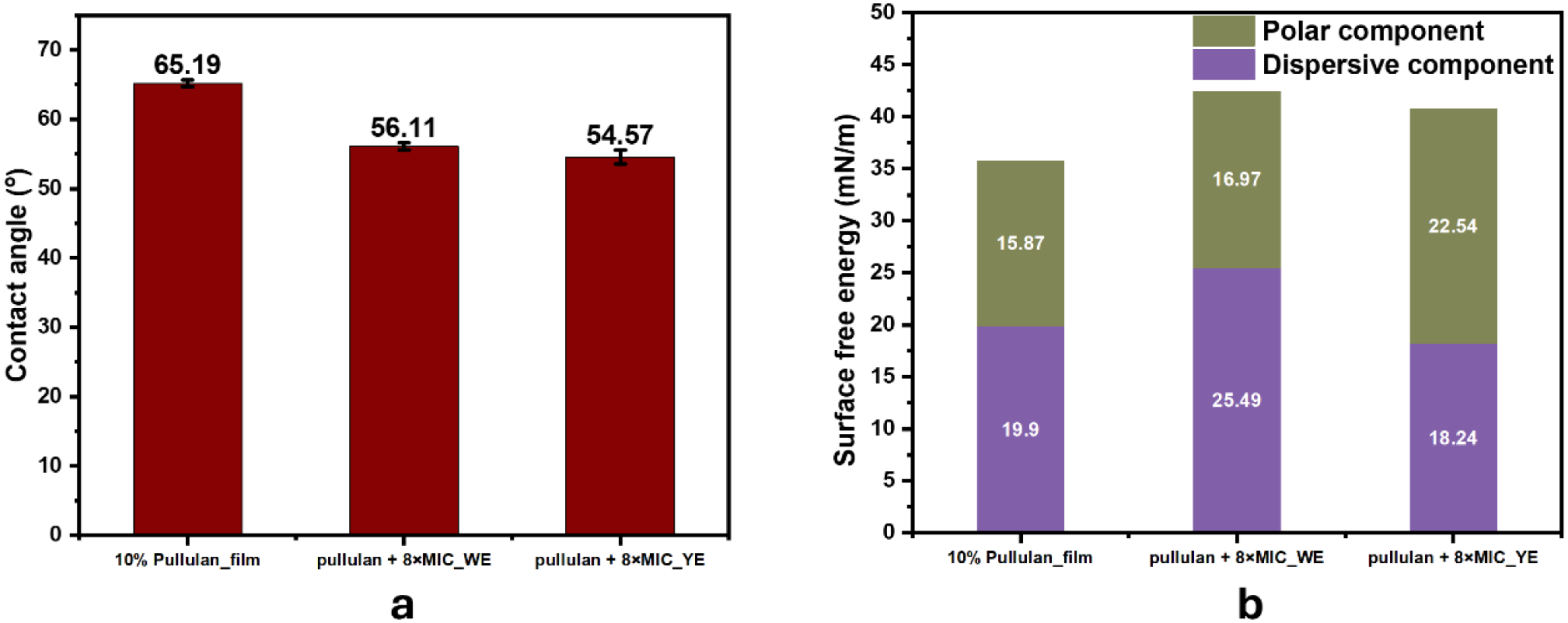
Surface properties of the cast films. a) contact angle and b) surface free energy.

##### 3.3.3.1 Contact Angle and Wettability

The neat pullulan film exhibited moderate water contact angles (65.19°), consistent with its polysaccharide structure, but indicative of limited surface wettability and adhesion potential, as reported previously for unmodified pullulan films.^15^ The incorporation of bioactive extracts reduced the contact angles, demonstrating enhanced surface hydrophilicity. The pullulan +8× MIC_WE film showed the lowest contact angles (56.10° for water), while the pullulan +8× MIC_YE film also exhibited reduced values (54.57° for water). These improvements are attributed to the introduction of polar functional groups from the phenolic constituents, such as gallotannins and ellagic acid in the WE and chlorogenic and caffeic acids in the YE, which promote surface–liquid interactions.

Enhanced wettability is advantageous for film forming coating applications, as it facilitates improved adhesion to substrates and more uniform coverage on the surface. Increased surface hydrophilicity may contribute to moisture spreading and antifogging behavior, which is beneficial for transparent food packaging materials.^24^

From an antimicrobial perspective, increased surface wettability also promotes closer and more intimate contact between the film forming coating on the packaging material surface and bacterial cells, thereby enhancing the effectiveness of contact-active antimicrobial agents embedded in the coating. Improved surface hydration can facilitate diffusion and availability of phenolic compounds at the interface, which is critical for inhibiting bacterial adhesion and growth.^79^

##### 3.3.3.2 Surface Free Energy (SFE)

The SFE values help predict adhesion behavior; a higher SFE generally improves the ability of films to spread on and adhere to different substrates.^80^ The pure pullulan film exhibited a total SFE of 35.77 mN/m with a dominant dispersive component $\left(\gamma_{s}^{D}\right)$ and minimal polar contribution $\left(\gamma_{s}^{P}\right)$ . This aligns with the expected behavior of nonfunctionalized polysaccharides, which interact primarily through van der Waals (dispersive) forces rather than strong polar or hydrogen-bonding interactions. Similar observations have been reported for other polysaccharide-based films, such as starch and chitosan, where the lack of significant polar groups results in low surface energy.^81^ The total SFE increased to 45.18 mN/m for the pullulan +8× MIC_WE film and 40.77 mN/m for the pullulan +8× MIC_WE film, indicating that both extracts enhanced the surface activity of the films.

The dispersive component $\left(\gamma_{s}^{D}\right)$ increased significantly with pullulan +8× MIC_WE film (25.49 mN/m), suggesting enhanced van der Waals interactions, likely due to aromatic π-systems in the tannins, like gallic acid, which contribute to the nonpolar interactions, while the YE (18.24 mN/m) maintained a similar level to neat pullulan. The $\gamma_{s}^{P}$ was highest in the pullulan +8× MIC_YE film (22.54 mN/m), reflecting the presence of polar compounds like chlorogenic acid, favoring hydrogen bonding and dipole interactions, potentially improving the adhesion and interfacial compatibility with polar substrates, while raising $\gamma_{s}^{D}$ moderately due to the minor aromatic content.

In summary, incorporating bioactive extracts into pullulan-based coatings modifies the surface free energy and wettability significantly, which are critical parameters influencing the coating performance. These modifications enhance adhesion to various food packaging substrates, particularly polymeric materials, by improving interfacial compatibility. Enhanced wettability facilitates better spreadability and uniform film formation during application, ensuring consistent coverage and functional performance.

#### 3.3.4 Optical Characteristics of Pullulan-Based Films

Exposure to ultraviolet (UV) and visible light can accelerate oxidative spoilage, nutrient degradation, and quality loss in packaged foods. Therefore, the UV–Vis transmittance of the films was analyzed, to assess their suitability as light-barrier materials for active packaging applications. The transmittance spectra of films prepared from 10% pullulan (plasticized with 5% glycerol) and its formulations containing 8× MIC_WE or 8× MIC_YE are shown in Figure 13.

**Figure 13:**
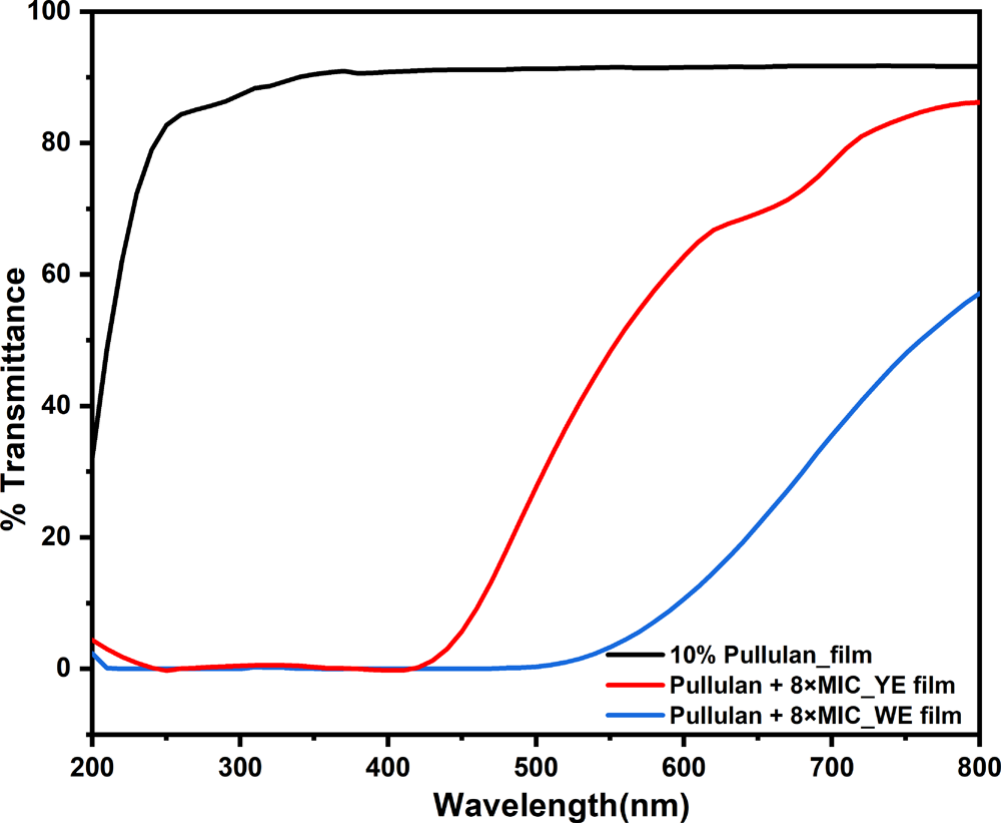
UV–Vis transmittance spectra of films.

The neat pullulan films exhibited high transmittance and minimal absorbance in the UV region (200–400 nm), confirming their limited intrinsic UV-blocking capability. This behavior is consistent with the high transparency of pullulan and its lack of UV-absorbing chromophoric groups.

In contrast, the extract-loaded films showed markedly enhanced UV-barrier performance. The pullulan +8× MIC_YE film displayed reduced transmittance across the UV range, particularly between 250 and 400 nm. This improvement is attributed to the presence of phenolic acids and flavonoids, such as chlorogenic acid, caffeic acid, and rutin, which possess high molar absorptivity in the UV region.^82^ Similarly, the pullulan +8× MIC_WE film exhibited the strongest UV-blocking effect, with a broader and steeper reduction in transmittance extending into the visible region (up to ∼600 nm), reflecting the high content of hydrolyzable tannins and aromatic phenolics characteristic of chestnut wood extracts.^37,83^

While partial attenuation in the visible range may reduce film transparency, this feature can be advantageous for packaging light-sensitive foods such as oils, dairy products, and functional beverages, where protection from photo-induced degradation is critical. Importantly, the extent of light shielding can be tuned through the extract selection and concentration to meet specific application requirements.^84^

These results confirm that the incorporation of WE and YE into a pullulan matrix enhances their UV-barrier performance significantly. The transmittance characteristics show that incorporating natural extracts can achieve a balance between UV shielding and visibility, which can be tuned according to the needs of the packaged food and consumer preferences. This functional property, combined with the improved physicochemical characteristics discussed earlier, positions these biobased films as promising candidates for active food packaging applications, offering protection against photooxidative spoilage, nutrient degradation, and quality loss.^8,85^

The color characteristics of the developed pullulan-based films were evaluated using the CIELAB color space, and the results are presented in Table 4.

**Table 4:** CIELAB Color Analysis Results

Sample	L*	a*	b*	C*	H	ΔL*	Δa*	Δb*	ΔC*	ΔE*	ΔH
10% Pullulan_film	72.40	–1.37	6.95	7.09	101.13						
Pullulan +8 × MIC_YE film	50.15	15.88	37.81	41.01	67.21	–22.26	17.25	30.85	33.92	41.77	–9.94
Pullulan +8 × MIC_WE film	27.00	6.00	6.63	8.94	47.85	–45.40	7.37	–0.33	1.85	46.00	–7.14

The incorporation of natural extracts altered the optical properties of the pullulan films markedly, as demonstrated by the CIELAB color analysis. The neat pullulan exhibited high lightness (L* = 72.40), low chroma (C* = 7.09), and a near-neutral hue (h = 101.13°), reflecting its transparent and colorless nature.

The addition of YE resulted in pronounced darkening and chromatic enhancement (L* = 50.15; ΔE* = 41.77), with substantial increases in redness and yellowness, yielding a saturated orange–yellow appearance (C* = 41.01; h = 67.21°). This strong color enrichment is attributed to the polyphenolic pigments inherent to yerba mate and is consistent with reports on extract-induced chromatic enhancement in biopolymer films.^46^ In contrast, WE incorporation led to the greatest reduction in lightness (L* = 27.00; ΔE* = 46.00), but only moderate chroma increase (C* = 8.94), producing a darker, reddish-brown film. The visual change in the WE-based films was dominated by intense darkening rather than hue saturation, likely due to the high tannin content.^86^

These distinct color responses indicate that YE is more effective in generating vibrant, saturated films, whereas WE contribute primarily to opacity and darkening. Both effects are visually significant (ΔE* > 40) and application relevant. YE-based films may be advantageous for intelligent or consumer-oriented packaging where visual cues or aesthetic appeal are desired, while WE-based films are better suited for light-protective packaging of UV-sensitive products such as oils and dairy.

The observed optical behavior is linked closely to the phenolic composition of the extracts. The aromatic structures and phenolic hydroxyl groups, confirmed by ATR-FTIR and HPLC analyses, enabled strong electronic transitions that absorb UV and visible light. Consequently, the extract-loaded films combined color modulation with effective photoprotection, contributing to reduced photo-oxidative degradation of the packaged foods. This strong color modulation observed in extract-loaded films is inherently linked to their functional phenolic content, indicating that the visual appearance of the films may serve as an indirect indicator of bioactive loading and protective performance.

In summary, natural extract incorporation enables tunable optical properties in pullulan films, allowing a balance between visual appearance and light-barrier performance. This versatility, combined with the elimination of synthetic colorants and UV stabilizers, reinforces the potential of these biobased films as sustainable, functional materials for tailored food-packaging applications.

#### 3.3.5 SEM Morphology

The SEM micrographs (Figure 14) reveal distinct differences in surface morphology among the films. The neat pullulan film exhibits a smooth, compact, and homogeneous surface, with no visible pores, cracks, or phase-separated domains.

**Figure 14:**
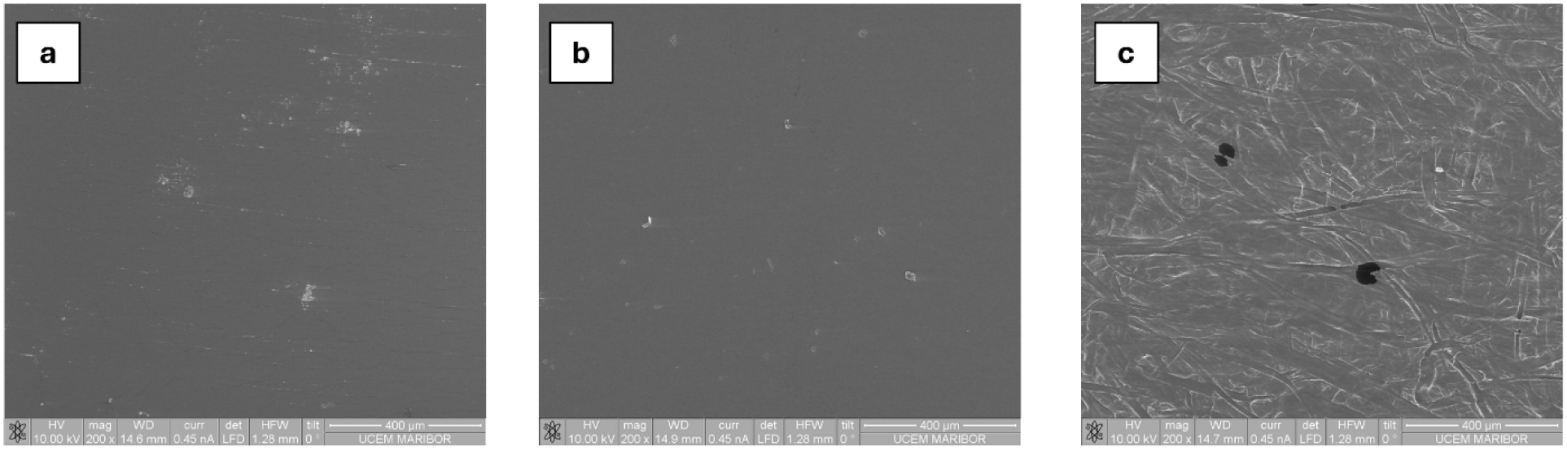
SEM micrographs of the films a) 10% Pullulan film b) Pullulan +8 × MIC_WE film and c) Pullulan +8 × MIC_YE film.

The pullulan +8× MIC_WE film shows a similarly smooth and uniform surface morphology, comparable to that of the neat pullulan film, with no evidence of large aggregates or surface discontinuities, indicating a homogeneous film structure at the micrometer scale.

In contrast, the pullulan +8× MIC_YE film displays a markedly different surface morphology, characterized by increased surface roughness, pronounced surface wrinkling, and the presence of fibrillar-like features and localized pores. Despite these microstructural features, the film remains continuous and free of macroscopic defects.

Overall, SEM analysis demonstrates that extract incorporation modifies the surface morphology of pullulan films in an extract-dependent manner, with YE leading to a more heterogeneous and structured surface compared to WE and neat pullulan films.

#### 3.3.6 XRD Structural Analysis

The XRD patterns of the neat pullulan and extract-loaded films (Figure S4) exhibited broad diffraction halos centered around 2θ ≈ 20°, confirming the predominantly amorphous nature of all the films. This amorphous structure is characteristic of pullulan-based materials and is favorable for flexible film formation and coating applications. Importantly, no sharp crystalline peaks were observed after the incorporation of YE or WE, indicating that the extracts were dispersed molecularly and did not induce crystallization or phase separation within the polymer matrix.

Subtle differences in diffraction intensity were evident between the formulations. The pullulan +8× MIC YE film showed a slightly increased diffraction intensity, suggesting enhanced molecular ordering or stronger intermolecular interactions. This observation correlates well with the ATR-FTIR results, indicating extensive hydrogen bonding, as well as with rheological data showing increased viscosity and shear-thinning behavior. Notably, this increased molecular organization is consistent with the more textured and fibrillar surface morphology observed by SEM, indicating that molecular-level interactions translate into microscale structural reorganization of the film surface.

In contrast, the pullulan +8× MIC_WE film exhibited marginally reduced diffraction intensity, consistent with the presence of tannin-rich components that may disrupt chain packing and exert a mild plasticizing effect. This interpretation aligns with the smoother and more homogeneous surface morphology observed in SEM micrographs, as well as with the more moderate changes in rheological behavior.

To summarize, XRD analysis confirmed that extract incorporation preserved the amorphous structure of the pullulan films while subtly modulating molecular organization through noncovalent interactions. When combined with SEM observations, these results demonstrate a clear structure hierarchy, where extract-dependent molecular interactions govern chain organization, which in turn dictates film microstructure and ultimately influences functional performance. This multiscale structure–property relationship supports the suitability of these systems for biobased coating applications.

#### 3.3.7 Antibacterial Activity

The antibacterial activity of the pullulan-based film forming coatings is summarized in Figure 15. The neat 10% pullulan showed no inhibition against *E. coli* or *S. aureus*, confirming the absence of intrinsic antibacterial activity. In contrast, the incorporation of yerba mate extract (YE) and chestnut wood extract (WE) enhanced the antimicrobial performance significantly.

**Figure 15:**
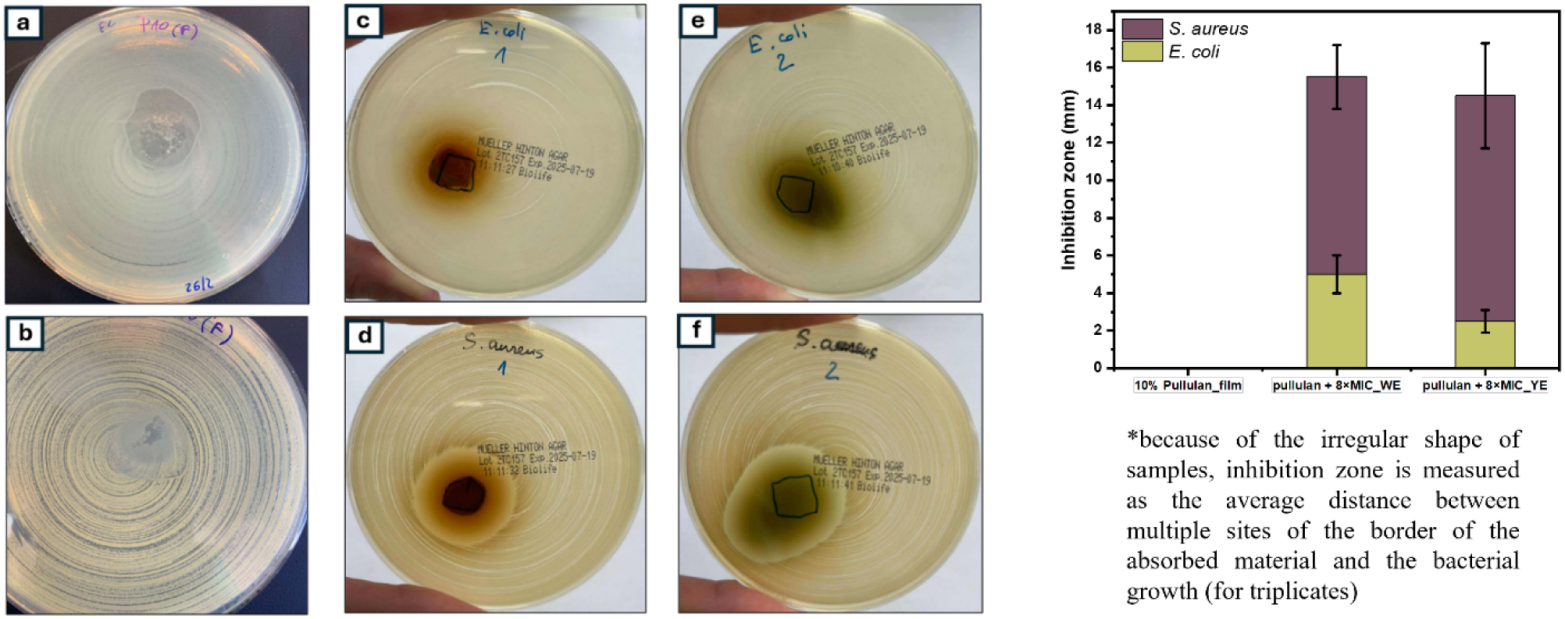
Antibacterial activity results of the cast films against *E. coli* (on the top) and *S. aureus* (on the bottom) respectively a), and b) 10% Pullulan film c) and d) pullulan +8 × MIC_WE film e) and f) pullulan +8 × MIC_YE film and the measured inhibition zone in mm. (on the left).

The YE-containing coating exhibited strong activity against *S. aureus* (12 ± 2.8 mm inhibition zone) and limited inhibition of *E. coli* (2.5 ± 0.6 mm), reflecting the selective efficacy of yerba mate polyphenols such as chlorogenic acids and flavonoids.^82,87^ The WE-based coating produced a comparable inhibition zone against *S. aureus* (10.5 ± 1.7 mm) and a moderate effect against *E. coli* (5 ± 1.0 mm), consistent with the known preference of tannin-rich extracts for Gram-positive bacteria. These trends align with previous reports demonstrating membrane disruption and oxidative stress induction as key antimicrobial mechanisms of polyphenolic compounds.^26,83^

The higher susceptibility of *S. aureus* compared to *E. coli* can be attributed to structural differences in the bacterial cell envelope. The absence of an outer lipopolysaccharide membrane in Gram-positive bacteria facilitates direct interaction between phenolic compounds and the cytoplasmic membrane, enhancing antimicrobial efficacy.^88^

The observed antibacterial behavior is supported by the surface and molecular characterizations discussed earlier. The ATR-FTIR and HPLC analyses confirmed the presence of aromatic and hydroxyl-rich phenolic structures, which are implicated directly in microbial membrane destabilization. Additionally, the extract-loaded coatings exhibited more negative zeta potential values and increased surface polarity compared to neat pullulan, factors known to reduce bacterial adhesion and initial colonization.^89^

The surface free energy analysis further revealed an increase in the polar component upon extract incorporation, resulting in more hydrophilic surfaces. Such surfaces are generally less favorable for bacterial attachment and can promote closer interfacial contact, facilitating the effective action of hydrophilic antimicrobial compounds at the coating–microbe interface. The combined contribution of passive antiadhesive effects and active bioactivity thus underlies the overall antibacterial performance of the coatings. Notably, the increased polar surface free energy, particularly in the pullulan +8× MIC_YE film, supports stronger interfacial interactions with microbial cell envelopes, which are rich in polar and charged functional groups. These interactions have been reported to enhance antimicrobial efficacy by promoting sustained contact and membrane destabilization in the presence of phenolic compounds. In summary, the extract-modified pullulan coatings demonstrated selective and effective antibacterial activity, particularly against *S. aureus*. This performance arises from the combined effects of phenolic bioactivity and surface-mediated contact mechanisms, rather than from reduced bacterial adhesion. The tunable antimicrobial response, governed by extract composition and interfacial properties, highlights the potential of these coatings for targeted food-packaging applications aimed at controlling specific microbial risks.^90^

Overall, the antibacterial activity of the extract-loaded pullulan films can be rationalized within the framework of extended DLVO theory, where interfacial interactions arise from a balance between electrostatic repulsion, van der Waals attraction, and acid–base (hydrogen-bonding) forces. Although both the film surface and *Staphylococcus aureus* cells carry a net negative charge at neutral pH, electrostatic repulsion does not preclude close contact. Instead, the presence of phenolic compounds introduces strong non-DLVO interactions, including hydrogen bonding and polar interactions, which lower the effective interaction energy barrier and enable intimate contact. This facilitates contact-active antimicrobial mechanisms such as membrane destabilization, particularly in Gram-positive bacteria lacking an outer lipopolysaccharide membrane.^91^

#### 3.3.8 Antioxidant Activity

The antioxidant performance of the films was evaluated using the DPPH radical scavenging assay, with visual confirmation provided by the ABTS radical discoloration (Figure 16a–b).

**Figure 16:**
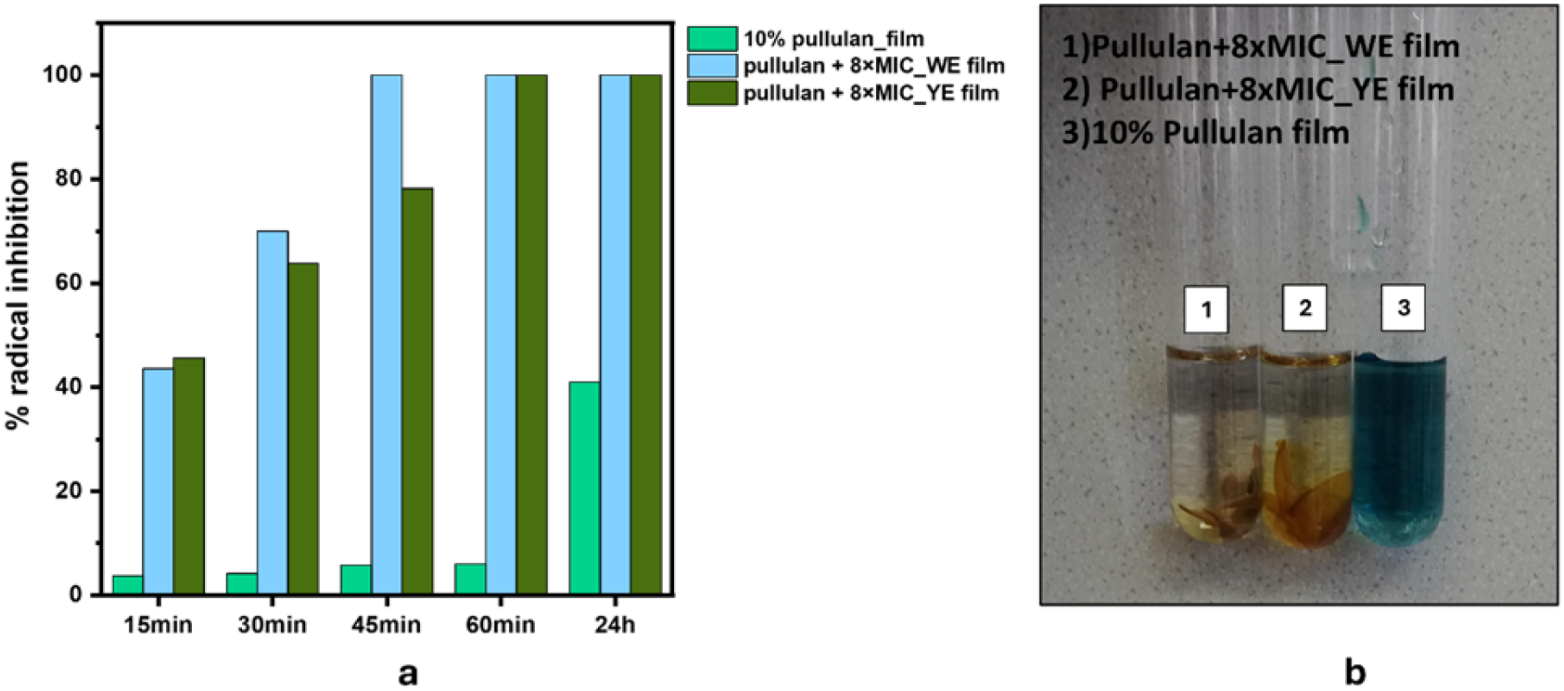
Antioxidative activity of the cast films. (a) DPPH assay results (b) image of discoloration of the ABTS radical assay.

In the DPPH assay (Figure 16a), both the extract-loaded films exhibited strong and sustained antioxidant activity, though with distinct kinetic profiles. The pullulan +8× MIC_YE film showed a rapid response, achieving complete radical inhibition within 45 min and maintaining this level for 24 h, indicating the high surface accessibility and reactivity of yerba mate phenolics. In contrast, the pullulan +8× MIC_WE film displayed a more gradual increase, reaching ∼80% inhibition at 45 min, reflecting the slower diffusion or lower reactivity of wood-derived phenolics, yet still demonstrating substantial scavenging capacity. The neat pullulan films showed only limited activity, reaching approximately 45% inhibition after 24 h, confirming that the antioxidant performance arises primarily from extract incorporation.

The antioxidant behavior of the films reflects the intrinsic radical scavenging capacity of the phenolic compounds identified by HPLC, such as chlorogenic, gallic, and ellagic acids, whose activity is well established in their pure form. The preservation of antioxidant efficacy across pure compounds, colloidal dispersions, and solid films indicates that the pullulan matrix acts as an inert carrier, enabling effective translation of molecular antioxidant functionality into coating-relevant formulations.

Notably, the antioxidant measurements were successful in the film state, unlike the pullulan dispersions that precipitated upon DPPH addition. This difference is attributed to the reduced polymer mobility and higher structural integrity of the films, which prevented aggregation and enabled stable interaction with radicals. The enhanced antioxidant performance of the extract-loaded films is supported further by the ATR-FTIR and surface free energy analyses, which confirmed the presence of phenolic hydroxyl groups and an increased polar surface component. These features promote hydrogen donation and electron transfer reactions, facilitating efficient radical neutralization.

The visual ABTS discoloration tests (Figure 16b) confirmed the quantitative results, showing rapid and pronounced fading for the extract-containing films, while neat pullulan produced only weak discoloration. Compared to the dispersions, the films exhibited slightly slower scavenging kinetics, which can be attributed to the matrix-controlled release of the phenolic compounds. Strong noncovalent interactions between the pullulan and phenolics, evidenced by the FTIR and SFE results, limited the antioxidant mobility temporarily, resulting in a gradual, yet complete release. Importantly, the pronounced antioxidant activity of the extract-loaded films complements their enhanced UV-blocking performance discussed previously. The same aromatic and conjugated phenolic structures responsible for UV absorption also act as efficient radical scavengers, providing a dual protection mechanism against photo-oxidative degradation. While UV shielding reduces the formation of light-induced reactive species, the antioxidant functionality actively neutralizes radicals that are generated within the packaged system, resulting in synergistic protection.

In summary, the extract-modified pullulan films provided effective and sustained antioxidant activity through a combination of active surface functionalization and controlled release from the polymer matrix. Together with the tunable UV-barrier properties, this dual functionality is particularly advantageous for active food-packaging applications, where prolonged protection against photo-oxidative spoilage and quality loss is required.

## 4 Conclusions

This study demonstrates a formulation-driven strategy for the development of pullulan-based colloidal coatings functionalized with natural polyphenol-rich extracts from yerba mate and chestnut wood, designed specifically for surface-applied active food-packaging systems. By shifting the focus from conventional bulk films to coating-relevant colloidal dispersions and their translation into solid films, this work establishes clear structure–property–function relationships across molecular, colloidal, interfacial, and solid-state levels.

The incorporation of YE and WE into pullulan matrices resulted in stable, homogeneous colloidal formulations with enhanced rheological behavior, controlled particle size, and favorable electrokinetic properties, enabling reproducible film formation. Upon solidification, the extract-loaded films exhibited pronounced antioxidant and selective antibacterial activity, effective UV-shielding, and tunable optical properties, while preserving the amorphous structure and mechanical integrity of the polymer matrix. Importantly, the bioactivity of the phenolic compounds was retained throughout the formulation and film-forming processes, confirming pullulan’s role as an inert yet highly effective carrier for natural functional agents.

Surface and interfacial analyses revealed that extract incorporation significantly modified wettability and surface free energy, particularly through increased polar contributions. These changes promote intimate interfacial contact and support contact-active antimicrobial and antioxidant mechanisms, rather than relying solely on passive barrier effects. At the same time, microstructural and structural analyses (SEM and XRD) demonstrated that extract-specific molecular interactions govern film morphology and organization, directly linking formulation chemistry to functional performance.

Beyond functionality, this work provides important insight into the coating applicability of pullulan-based systems, highlighting how colloidal stability, surface activity, and film continuity can be tuned through extract selection without compromising sustainability. The ability to modulate surface energy, optical response, and bioactivity within a fully biobased system underscores the adaptability of these coatings for integration into multilayer packaging concepts, where interfacial compatibility and controlled functionality are critical.

Overall, this study presents a scalable and sustainable platform for multifunctional biobased coatings, combining antioxidant, antimicrobial, and UV-protective performance with coating-relevant physicochemical properties. By bridging formulation science with surface and film characterization, the work advances pullulan–polyphenol systems as promising candidates for next-generation active food-packaging technologies that address both food safety and environmental sustainability.

## Supplementary Material



## Notes

Minimal inhibitory concentrations (MIC) of the extracts (Table S1); total phenolic content and extraction parameters (Table S2); identification and quantification of phenolic compounds in yerba mate and chestnut wood extracts by HPLC (Tables S3–S4) with corresponding chromatograms (Figures S1–S3); and XRD patterns of neat pullulan and extract-loaded films (Figure S4) (PDF)
