# Supplementary material for: Phenolic-Enriched Pullulan Coatings: Molecular Interactions and Functional Properties for Active Food Packaging Applications
Source: ACS Omega. 2026 Mar 2;11(10):16854–75. doi: 10.1021/acsomega.6c00375 (PMC13000653; doi:10.1021/acsomega.6c00375)
Supplement: Supplementary file 1 [file ao6c00375_si_002.pdf]

Supplementary Data for:

Phenolic-Enriched Pullulan Coatings: Molecular Interactions and Functional Properties for Active Food Packaging Applications

Athira John [a*], Klementina Pušnik Črešnar [a, b], David Hvalec [b], Maša Knez Marevci [b, c], Dimitrios N. Bikiaris [d], Lidija Fras Zemljič [a*]

[a] Faculty of Mechanical Engineering, University of Maribor, Smetanova Ulica 17, 2000 Maribor, Slovenia

[b] Faculty of Chemistry and Chemical Engineering, University of Maribor, Smetanova 17, 2000 Maribor, Slovenia

[c] Faculty of Medicine, University of Maribor, Taborska Ulica 8, 2000 Maribor, Slovenia

[d] Laboratory of Polymer Chemistry and Technology, Department of Chemistry, Aristotle University of Thessaloniki, 541 24, Thessaloniki, Macedonia, Greece

Table S1. Minimal inhibitory concentrations of the extracts.

Sample	MIC [mg/mL]		
	Staphylococcus aureus	*Escherichia Coli*	*Candida Albicans*
Yerba	9.38	9.38	9.38
Wood	4.69	9.38	9.38

Table S2. Total phenolic content of the extracts.

Method	Material	Solvent	m_0 [g]	β [%]	wGAE [mg/g]
Sonification	WE	Water	20.02	7.7053	579.2622
Sonification	YE	Water	20.022	29.1180	238.6268
-	Pullulan	Water	20.02	0	0

m_0 [g]	Mass of initial material for extraction
β [%]	Yield of extraction
wGAE [mg/g]	Amount of total phenols in gallic acid equivalent

Table S3. Phenolic compounds in yerba mate extracts determined by HPLC.

Compound [µg/mg]	Chlorogenic acid	Caffeic acid	p-coumaric acid	Rutin
YE	42.11	0.45	Tr.*	21.64

*Tr - traces

Table S4. Phenolic compounds in wood extracts determined by HPLC.

Compound [µg/mg]	Gallic acid	Epicatechin	Ellagic acid
WE	28.56	Tr*	42.09

*Tr - traces

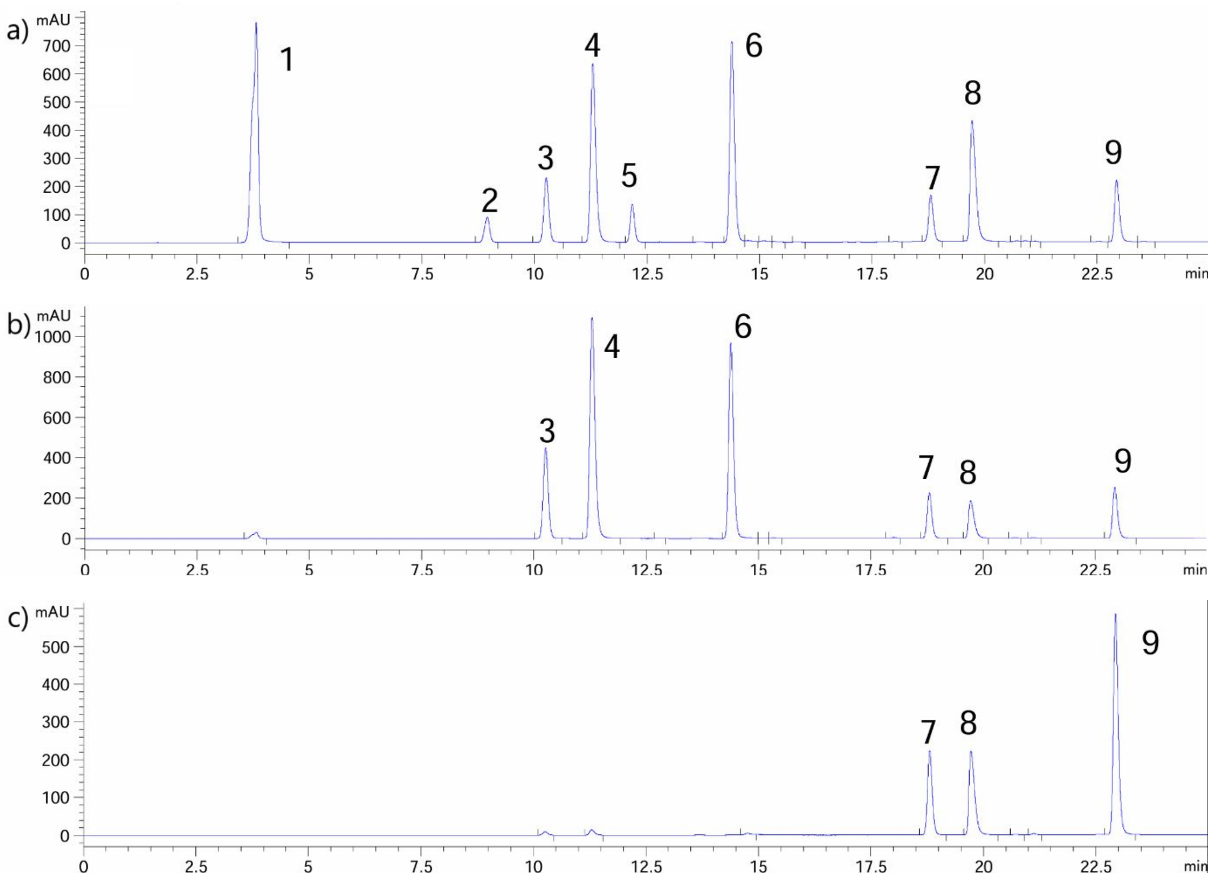

Figure S1. HPLC chromatograms for a mixture of standards (1, gallic acid; 2, catechin; 3, chlorogenic acid; 4, caffeic acid; 5, epicatechin; 6, p-cumaric acid; 7, rutin; 8, ellagic acid; 9, quercetin) at a) 280 nm, b) 320 nm and c) 380 nm.

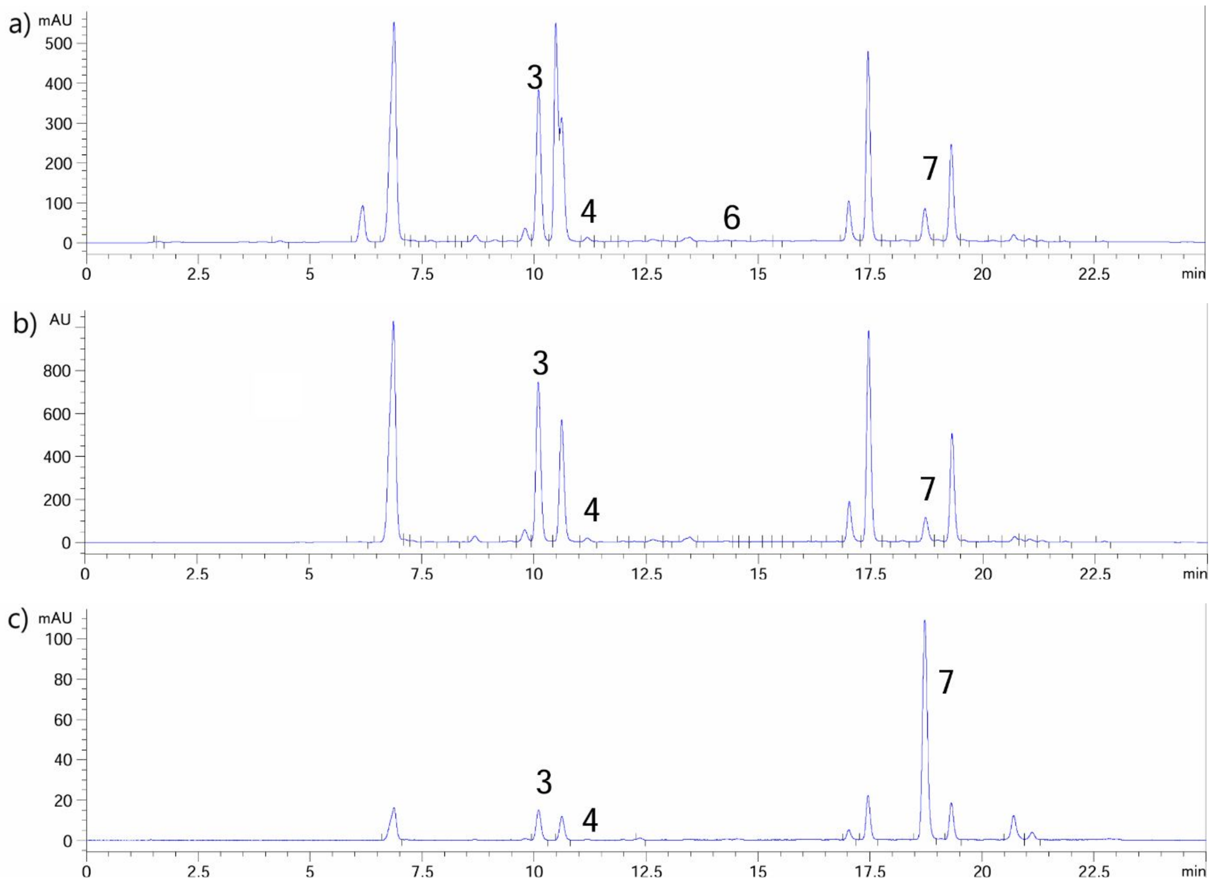

Figure S2. HPLC chromatograms for the determination of polyphenolic compounds (3, chlorogenic acid; 4, caffeic acid; 6, p-cumaric acid; 7, rutin) in yerba mate extract at a) 280 nm, b) 320 nm and c) 380 nm.

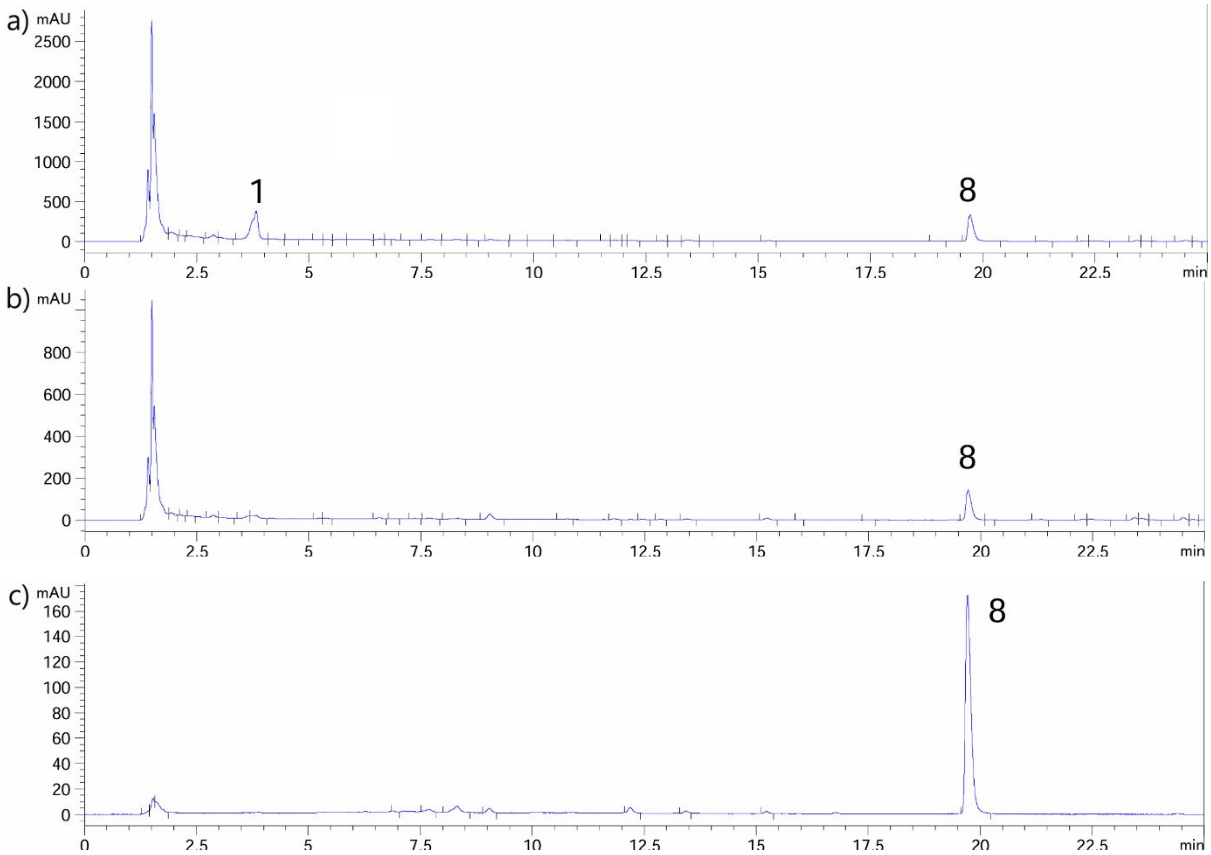

Figure S3. HPLC chromatograms for the determination of polyphenolic compounds (1, gallic acid; 8, ellagic acid) in chestnut wood extract at a) 280 nm, b) 320 nm and c) 380 nm.

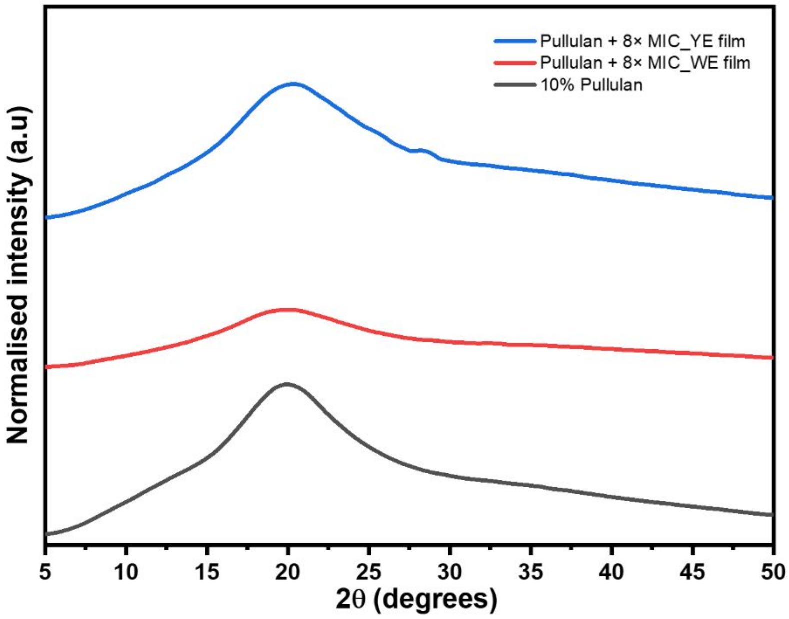

Figure S4. XRD patterns of neat pullulan and extract-loaded films
